# Model-based projections for COVID-19 outbreak size and student-days lost to closure in Ontario childcare centres and primary schools

**DOI:** 10.1038/s41598-021-85302-6

**Published:** 2021-03-18

**Authors:** Brendon Phillips, Dillon T. Browne, Madhur Anand, Chris T. Bauch

**Affiliations:** 1 Department of Applied Mathematics, University of Waterloo, Waterloo, ON Canada; 2 Department of Psychology, University of Waterloo, Waterloo, ON Canada; 3 School of Environmental Sciences, University of Guelph, Guelph, ON Canada

**Keywords:** Computational models, Ecological modelling, Population dynamics, Biological physics, Computational science, Infectious diseases, Respiratory tract diseases

## Abstract

There is a pressing need for evidence-based scrutiny of plans to re-open childcare centres during the COVID-19 pandemic. Here we developed an agent-based model of SARS-CoV-2 transmission within a childcare centre and households. Scenarios varied the student-to-educator ratio (15:2, 8:2, 7:3), family clustering (siblings together versus random assignment) and time spent in class. We also evaluated a primary school setting (with student-educator ratios 30:1, 15:1 and 8:1), including cohorts that alternate weekly. In the childcare centre setting, grouping siblings significantly reduced outbreak size and student-days lost. We identify an intensification cascade specific to classroom outbreaks of respiratory viruses with presymptomatic infection. In both childcare and primary school settings, each doubling of class size from 8 to 15 to 30 more than doubled the outbreak size and student-days lost (increases by factors of 2–5, depending on the scenario. Proposals for childcare and primary school reopening could be enhanced for safety by switching to smaller class sizes and grouping siblings.

## Introduction

As nations around the world grapple with the psychosocial, civic and economic ramifications of social distancing guidelines^1,2^, the critical need for widely-available Early Childhood Education (or colloquially, “childcare”) services have once again reached the top of policy agendas^3,4^. Whether arguments are centred on human capital (i.e., “children benefit from high-quality, licensed educational environments and have the right to access such care”) or the economy (i.e., “parents need childcare in order to work, and the economy needs workers to thrive”), the conclusion is largely the same: childcare centres are re-opening (at least in some capacity) and this is taking place before a vaccine or herd immunity can mitigate the potential spread of SARS-CoV-2 (the virus that causes COVID-19). Outbreaks of COVID-19 in emergency childcare centres and schools have already been observed^5^, causing great concern as governments struggle to balance “flattening the curve” and preventing second waves with other pandemic-related sequelae such as the mental well-being of children and families, access to education and economic disruption.

Governments and childcare providers are tirelessly planning the operations of centres, with great efforts to follow public health guidelines for reducing SARS-CoV-2 contagion^6^. However, these guidelines, which will result in significantly altered operational configurations of childcare centres and substantial cost increases, have yet to be rigorously examined. Moreover, discussions of childcare are presently eclipsed by general discussion of school reopening^7^ and the harms versus benefits of school closure during a pandemic^8,9^. That being said, for many parents, the viability of the school-day emerges from before- and after-school programming that ensures adequate coverage throughout parents’ work schedules. Yet, reopening plans often fail to mention the critical interplay between school and childcare, even though many childcare centres operate within local schools^10^. Consequently, a model that comprehensively examines the multifaceted considerations surrounding childcare operations may help inform policy and planning. As such, the purpose of the present investigation is to develop an agent-based model that explores and elucidates the multiple interacting factors that could impact potential SARS-CoV-2 spread in school-based childcare centres.

In Ontario, Canada, childcare centres were permitted to reopen on June 12, 2020, provided they limit groupings (e.g., classrooms) to a maximum of 10 individuals (educators and children, inclusive)^11^. Additionally, all centres had to come up with a plan for daily screening of incoming persons, thorough cleaning of rooms before and during operations, removal of toys that pose risk of spreading germs, allowing only essential visitors, physical distancing at pick-up and drop-off, and a contingency plan for response should anyone be exposed to the virus (e.g., closing a classroom or centre for a period of time). Further school-specific recommendations have been recently outlined by The Toronto Hospital for Sick Children^10^, which include specific guidelines for screening, hand hygiene, physical distancing, cleaning, ventilation, and masking. While this influential report has become the guiding framework for school reopening in Ontario, there remains no discussion of childcare operations in relation to SARS-CoV-2 spread. Guidelines for primary schools call for either full re-opening with up to 30 students per classroom attending every day, or with cohorts of 15 students attending in alternate weeks. Most recently and at the time of resubmission of this paper, Ontario has reported COVID-19 test positivity rates in schoolchildren that are twice that of other ages (approximately $16 \%$ versus $8 \%$) and schools have been closed for a second time during the pandemic in order to minimize COVID-19 transmission^12^.

Simulation models of infectious disease spread have been widely applied during the COVID-19 pandemic, as in previous pandemics^13,14^. Modelling is used to determine how quickly the pathogen can spread^15^, how easily it may be contained^16^, the relative effectiveness of different containment strategies^17,18^, the social and economic impacts of lockdown^1,2^, and the role of schools in transmission^19,20^. Sensitivity analysis is crucial to assess whether model predictions are robust to uncertainties in data^21^, which is particularly important during a pandemic caused by a novel emerging pathogen like SARS-CoV-2. Agent-based models are particularly well-suited to situations where a highly granular description of the population is desirable and where random effects (stochasticity) are important. Such modelling has been used in both pandemic and non-pandemic contexts^22–24^, and is the basis of our methodology in the present work focusing on SARS-CoV-2 transmission in schools and households. Our objective is to use agent-based modelling to project the impact of student-educator (or in the case of childcare centres, child–educator) ratios and sibling grouping strategies on outbreaks of COVID-19 and student-days lost to classroom closure in a hypothetical childcare centre and primary school.

## Model overview

**Figure 1:**
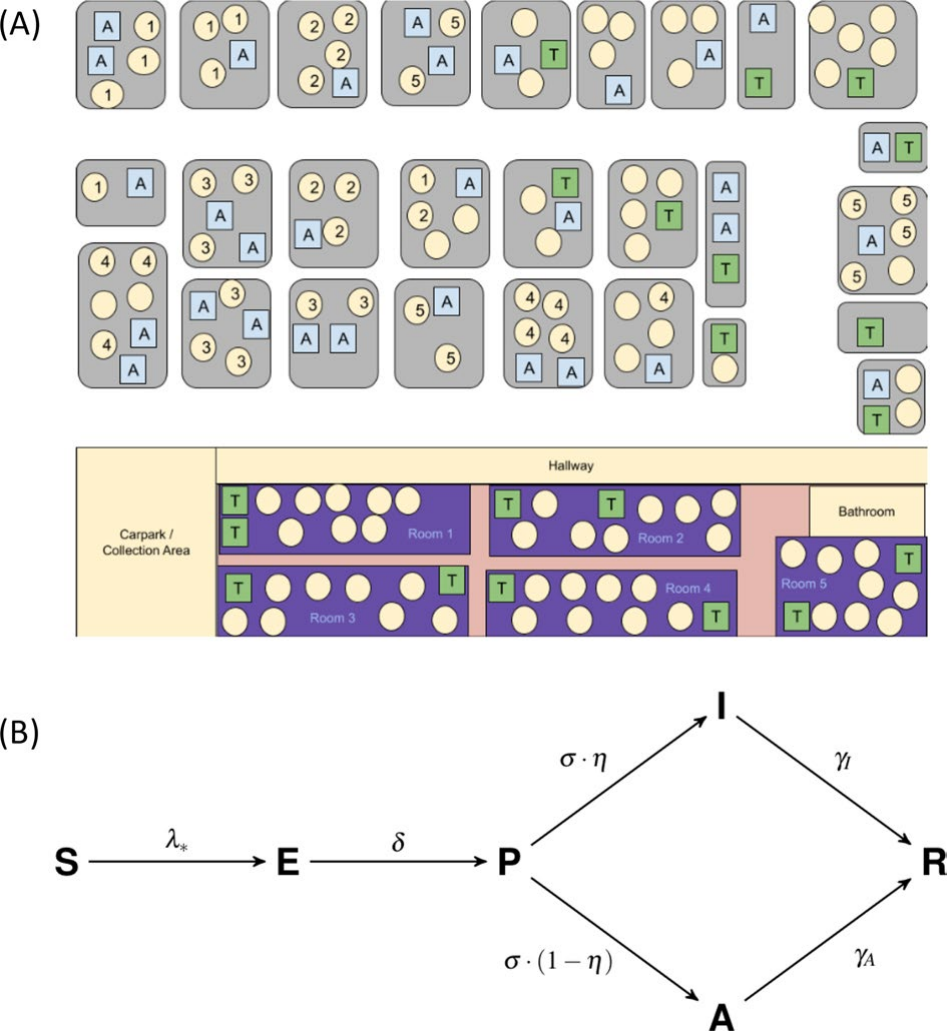
(**A**) Schematic representation of model population. ‘A’ represents adult, ‘T’ represents educator, and circles represent children. Grey rectangles represent houses and the school is represented at the bottom of the figure. Numbers exemplify possible assignments of children in households to classrooms. (**B**) Diagram showing the SEPAIR infection progression for each agent in the simulation (see Methods for definitions of parameters).

A detailed description of the model structure, assumptions and parameterisation appears in the “Materials and methods” section. We developed an agent-based model of SARS-CoV-2 transmission in a population structured by household and classroom groupings, as might represent a childcare setting or a small primary school (Fig.  1a). Individuals were categorized as either child or adult, and contacts between these groups were parameterised based on contact matrices estimated for the Canadian setting. Household sizes were determined from Canadian demographic data, with classroom sizes and student-educator ratios determined according to the scenario being studied. For the childcare setting we analyzed student-educator ratios of 8:2 and 7:3, giving a maximum class size of 10 (representative of smaller enrollment at schools). We also analysed a student-educator ratio 15:2 (giving a total class size of 17) as well as classroom assignment. Individuals may spread the infection to their household members each day, so that various patterns of effective contacts and interaction in the classroom may result in qualitatively different spreading patterns. As such, children in this model can be assigned to classrooms either randomly (*RA*) or by grouping siblings (or otherwise cohabiting students) together (*ST*) in an attempt to reduce SARS-CoV-2 transmission. For the primary school setting, we considered student-educator ratios of 8:1, 15:1 and 30:1 with all students randomly assigned. For the 8:1 and 15:1 ratios, we also considered scenarios where cohorts of 8 or 15 students attend the same classroom in alternate weeks, labelled 8(A):1 and 15(A):1 respectively. In the primary school setting, we considered the higher student-educator ratio 30:1 as an example of larger class size. Some plans considered in reopening Ontario educational institutions divides this larger class size into two alternating cohorts of 15 students each with a single shared educator; we also label this scenario 15(A):1. Rotation occurs each week, so that one cohort engages with online material while the other receives face-to-face instruction for 5 days, after which the cohorts exchange roles. The student-educator ratios 8:1 and 8(A):1 were also included for comparison to smaller class sizes. For primary schools we considered only the RA assignment.

SARS-CoV-2 can be transmitted in households, classrooms, or in common areas of the school, all of which were treated as instances of homogeneous mixing on account of evidence for aerosolized routes of transmission^25^. Individuals were also subject to a constant background risk of infection ($\lambda _*$) from other sources, such as shopping centres. Figure 1b shows the progression of the illness experienced by each individual in the model. In each day, susceptible (*S*) individuals exposed to the virus via community spread or interaction with infectious individuals (those with epidemiological statuses *P*, *A* and *I*) become exposed (*E*), while previously exposed agents become presymptomatic (*P*) with probability $\delta$. Presymptomatic agents develop an infection in each day with probability $\delta$, where they can either become symptomatically infected (*I*) with probability $\eta$ or asymptomatically infected (*A*) with probability $1-\eta$. If a symptomatic individual appears in a classroom, that classroom is closed for 14 days (in the case of alternating cohorts for primary schools, neither cohort attends class during this period); other classrooms in the same school may remain open during this time. Asymptomatic students and educators return at the end of this 14-day period while symptomatic students and educators remain at home, with symptomatic educators being replaced by substitutes.

Children are less affected by the SARS-CoV-2 virus than adults, and account for a smaller proportion of COVID-19 cases^26^. However, the role of children in SARS-CoV-2 transmission is still debated, and existing epidemiological evidence is limited by lack of empirical studies in school settings, which have been closed for much of 2020. Other studies show that children shed a similar amount of virus to adults^27^. To account for this ambiguity, we used contact matrices drawn from populations under ‘business as usual’ circumstances as a proxy of what contact rates would look like under a full reopening of schools and workplaces^28^. To account for the use of safety measures within the school (and the compliance of children and teachers with best practices), we consider low and high transmission rate scenarios (as seen in Supplementary Tables S1 and S2). The low transmission rate scenario represents reduced transmission rates in children (vs. adults) and/or highly effective infection control through consistent use of high-effectiveness masks, physical distancing and disinfection protocols (see “Methods” section for details). Similarly, the high transmission rate scenario represents inconsistent use (or non-use) of masks, ignoring distancing guidelines, improper hygiene and all other factors facilitating disease spread. To see the effect of shorter schooling durations, we also consider a ‘reduced time’ scenario in which students spend less time in class. In total, the permutations on student-educator ratios, transmission rate assumptions, siblings versus non-sibling groupings, alternating cohorts and schooling duration yielded a number of scenarios detailed in Supplementary Tables S1 and S2.

## Results

We first discuss the results obtained for childcare centre models, marked by class sizes varying between 10 and 17 (student-teacher rations 7:3, 8:2 and 15:2), different classroom assignment schemes and school day durations. Primary schools, on the other hand, feature larger class sizes (student-teacher ratios 8:1, 15:1, 30:1), assignment at random and possible division of students into weekly alternating cohorts. The results of simulations with the primary school model are detailed in the “Primary School settings” section and the Supplementary Information. The code used is available here.

### Initial stages of the outbreak

The time evolution of the outbreaks are illustrated in Supplementary Fig. S1, which shows the proportion of actively infected school attendees (both children and educators) per day in twelve childcare centre scenarios. Many of the scenarios tend to produce a well-defined outbreak curve close to the start of the simulation, even with classroom closure protocols in place. However, the outbreaks are more strongly household-driven for the 7:3 and 8:2 ratios than the 15:2 ratio; this is apparent in the weekly waves superimposed on the overall epidemic curve more strongly in the 15:2 scenarios on account of the impact of weekends. The 15:2 ratio also tends to generate earlier, more intense outbreaks, while 7:3 and 8:2 student-educator ratios produce fewer infections that are more sporadically distributed throughout the simulated time horizon. In the case of high transmission, the maximum mean level of exposure (*E*) is $5.03\%$ in the 15:2 RA scenario (on average) 12 days into the the simulation, with peak $3.18\%$ presymptomatic (*P*) and $1.63\%$ asymptomatic (*A*) proportions of attendees at days 12 and 19 respectively. Meanwhile, peak mean exposure in scenario 7:3 ST occurs on day 2, with $2\%$ attendees exposed to the disease and presymptomatic cases never exceeding that of the start of any simulation.

Supplementary Table S3 shows the 30-day peak value of each proportion of active infections in the childcare centre and the times at which these peaks occur (given in days). Here we can see that active infections peak far earlier with the ST assignment than with the RA assignment for both high ($\alpha _C=0.75$) and low ($\alpha _C=0.25$) transmission rates in most cases, with either equal or smaller peaks for most maximum proportions corresponding to the RA assignment independent of student-educator ratio. In the case of high transmission, peak proportions decrease with the number of students per class in half of the tested scenarios (statuses *P* and *I* with RA assignment, and status *E*). In the low transmission case, there is a reversal in trend, with peak proportions increasing with decreasing number of exposed (*E*) and presymptomatic (*P*) students per class. There is no obvious relationship between peak days for infected (*I*) and asymptomatic (*A*) individuals in the high transmission case, neither for asymptomatic (*A*) individuals in the low transmission case.

**Figure 2:**
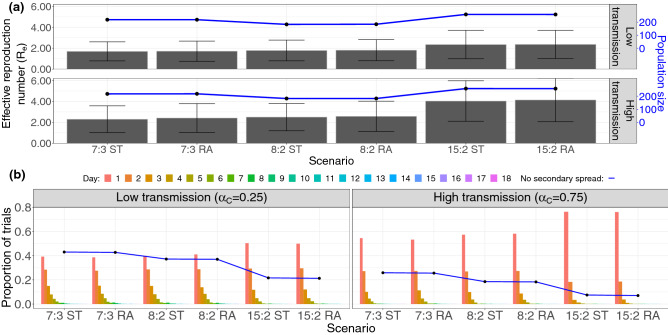
Diagrams showing the characteristics of COVID-19 spread in the childcare context. (**a**) Bar chart showing the effective reproductive number $R_e$ of the entire population (with error bars denoting one standard deviation), with a line plot showing the mean population size. For this childcare centre model, both low and high transmission scenarios are shown. (**b**) Diagram showing the proportion of trials without secondary spread (curve) in the childcare centre and the time taken to produce the first secondary infection (bar charts), both sorted by scenario.

The basic reproduction number $R_0$ is the average number of secondary infections produced by a single infected person in an otherwise susceptible population^29^. When there is pre-existing immunity (as we suppose here), we study the effective reproduction number $R_e$ - the average number of secondary infections produced by a single infected person in a population with some pre-existing immunity. Figure 2a shows the estimated effective reproduction number $R_e$ and mean population size (the number of individuals in all households occurring in the model) over the course of each simulation, computed by tracking the number of secondary infections produced by a single primary case. The $R_e$ values measured from the simulation range from 1.5 to 4 on average, depending on the scenario. These $R_e$ values encompass the typical range of $R_0$ values reported in the literature^30^. This is the expected relationship, not only because of pre-existing immunity, but also because the $R_e$ values in our simulation capture transmission only in schools and workplaces, while the $R_0$ values in the literature are measured for SARS-CoV-2 transmission in all settings, including workplaces and other sources of community spread.

There is little correlation between mean population size (Fig. 2a, line), number of households (not shown) and the corresponding $R_e$ estimate (Fig. 2a, bars), leaving only the number of children per classroom responsible for the gross increasing trend in $R_e$ in both the high ($\alpha _C=0.75$) and low ($\alpha _C=0.25$) transmission scenarios. Equation 3 shows that child–child contact within the classroom occurs at least 2 times more often than any other type of contact; given that the majority of the attendees of the school are children, we can expect $R_e$ to depend on the number of children enrolled in the school.

This is further demonstrated by the bar charts of Fig. 2b, which show the distribution of times between the primary infection case and the first secondary infection. The scenario with the highest student-educator ratio (15:2) shows both the greatest propensity for disease outbreak (that is, possessing the highest proportion of trials with secondary spread) and the quickest start of the outbreak in both high and low transmission cases, with 15:2 RA having the highest proportion of trials where the first secondary infection occurred within a single day, in the high transmission case. In comparison, scenario 7:3 RA showed the slowest average initial spread in the high transmission case, while the low transmission case sees low rates for both 8:2 and 7:3. Assigning siblings to the same classroom (ST) frequently results in faster secondary spread over the first 2 days (even in the first 2 weeks) in the 8:2 and 15:2 student-educator scenarios.

### Outbreak duration

**Figure 3:**
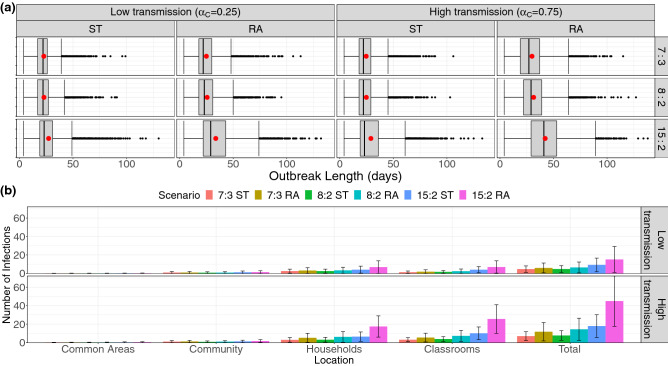
Diagrams showing the outbreak length in the population and the number of exposures in the childcare centre setting. (**a**) Box plots depicting the distribution of simulation durations for each scenario. Taken together with the stopping criteria of the simulations and measures of aggregate, these describe the duration of the outbreak in a district hosting a childcare centre. Red dots denote the mean outbreak length (that is, mean simulation length). (**b**) The mean number of infections occurring among all childcare centre attendees in each location over time for each scenario. The height of each bar gives the ensemble mean and its standard deviation is represented by error bars.

Each individual simulation ends when all classes are at full capacity and there are no active infections in the population; aside from community infection, this marks the momentary halt of SARS-CoV-2 spread. From this, we get a description of the duration of the first outbreak (there could well be a second outbreak sparked by some community infection among individuals who remain susceptible at the end of the first outbreak). Box plots in Fig. 3a show that the 15:2 ratio in both RA and ST assignments gives a median outbreak duration at least as large as all other scenarios (for both low and high transmission cases). Another general observation is that classroom assignment (RA vs. ST) doesn’t change the distribution of outbreak duration for student-educator ratios 8:2 and 7:3 as drastically as it does for 15:2, whereas ST assignment results in lower median duration (24 vs 43 for RA assignment) and significantly lower maximum duration for the 15:2 ratio (61 versus 88 for RA assignment without outliers) in the high transmission case.

This is mirrored in the low transmission case as well. A possible explanation lies in the number of students per classroom. The child–child contact rate (Eq. 3) is far higher than any other contact rate, implying that the classroom is the site of greatest infection spread (demonstrated in Fig. 3b, where there is more infection occurring in the classroom that in any other location in every scenario). ST assignment differs from RA assignment in its containment of disease transfer from the classroom to a comparatively limited number of households. This effect (the difference between ST and RA assignment) is amplified with the addition of each new student to the classroom, so that while the difference between 7:3 and 8:2 may be small (only 1 student added), the effect becomes far exaggerated when the student number is effectively doubled (15 students vs. 7 or 8).

**Figure 4:**
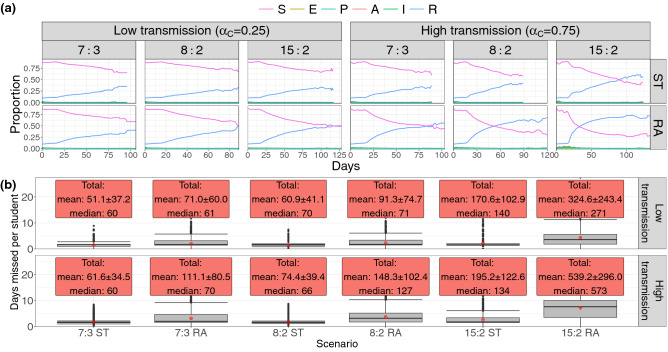
Plots detailing the trends in outbreak progression through the simulation, and the number of student-says missed in the childcare centre setting, both per-student and total. (**a**) Time series detailing the trends in the mean proportions of current childcare centre attendees in each stage of disease progression. Larger class sizes result in more infections in total, and more infections occur in classrooms than any other locations. Shaded ribbons around each curve show one standard deviation of the averaged time series. Only trials showing secondary spread were included in the ensemble means shown. (**b**) Box plots demonstrating the mean number of face-to-face days missed per student over the course of the simulation due to class closure upon the detection of an outbreak in the childcare centre setting. Text boxes give the means, standard deviations and medians of the total number of days missed by all students in each simulations.

The evolution of the numbers of susceptible (*S*) and recovered/removed (*R*) childcare centre attendees provides additional information on the course of the outbreak, since they represent the terminal states of the infection process in each individual by the end of the outbreak. Figure 4a shows the proportion of susceptible and recovered current school attendees (who have not been sent home due to classroom outbreaks). As with all results so far, the 15:2 RA scenario most efficiently facilitates disease spread through the centre in both high and low transmission cases, with the proportion of recovered attendees (*R*) overtaking the number of never-infected attendees (status *S*) on day 34 in the case of high transmission ($\alpha _C=0.75$). Performance between 8:2 and 7:3 ratios with ST assignment is similar for both transmission rates, though all scenarios show smaller variation over trials featuring lower infection transmission. As shown in Fig. 3a, scenario 15:2 RA gave the longest average simulation time in the high transmission scenario; this is also reflected in Fig. 4, where the longest outbreak lasted 138 days.

### Outbreak size and classroom closure

Figure 3b shows the mean number of infections in each location of the childcare centre in all scenarios, as well as the total number of infections produced in each scenario (the ‘outbreak size’). As expected, many more infections occur in the high transmission scenario ($\alpha _C=0.75$), and the error bars of the plot show greater standard deviation of the results than in the low transmission ($\alpha _C=0.25$) scenario. However, for each location, and regardless of the transmission rate scenario, the number of infections increases rapidly with the number of children in the classroom in each room assignment scheme. The 15:2 ratio is universally the worst ratio across all childcare centre scenarios tested. However, the difference between the outbreak size in different scenarios decreases as the transmissibility of the virus drops (so to speak, the gap been between the 15:2 RA and 15:2 ST scenarios decreases as $\alpha _C$ decreases, and so with other student-educator ratios). When the transmission rate is high, the relatively larger variety (by household) and prevalence of child–child interactions has a multiplicative effect on the number of effective transmissions in the classroom. Lower transmissibility thereby decreases the classroom infection rates relative to the household transmission rates.

The numbers of student-days missed due to outbreak-driven classroom closure (per student and total) in the childcare centre are given in Fig. 4b. For each enrolled child, a single day of classroom closure is counted as a missed student-day if the child would have otherwise been in class, that is, any weekday on which the child shows no symptoms). In all scenarios, the 15:2 student-educator ratio is quantitatively the worst strategy examined by almost an order of magnitude, resulting in both the highest possible number of student-days missed as well as an increase in the mean number of student-days lost per individual student (that is, normalizing by the number of students in the centre). Here, RA assignment shows worse performance than ST assignment in all scenarios. Both the low ($\alpha _C=0.25$) and high ($\alpha _C=0.75$) transmission scenarios favour the 7:3 student-educator ratio and ST assignment, with a lower number of student-days missed. The poor performance of 15:2 ratio occurs because it suffers from a multiplicative effect: larger class sizes are more likely to be the origin of outbreak, and when the outbreak starts, more children are affected when the classroom is shut down. Moreover, since it’s possible for a student or educator to be infected during a 14-day closure, not all attendees necessarily return to class upon reopening; sick educators are replaced with substitutes, while ill students simply remain at home. As such, these class closures result in otherwise healthy students missing potentially additional school days beyond the 14-day closure period. The 15:2 strategy suffers particularly from this effect, since transmission is further facilitated when more students are in a classroom.

### Primary school settings

**Figure 5:**
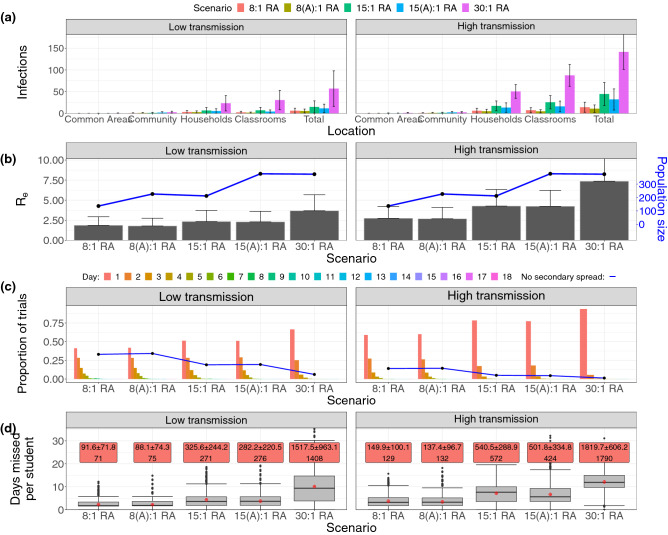
The effective reproduction number, location-specific numbers of exposure and numbers of missed student-days in various scenarios in the primary school setting. (**a**) Bar charts showing the mean number of infections occurring in households and each location of the primary primary school over the course of the simulation. (**b**) Bar charts showing the effective reproductive number $R_e$ in a population hosting a primary school, with error bars denoting one standard deviation. Both low and high transmission scenarios are shown. (**c**) Diagram showing the proportion of trials without secondary spread (line) and the mean time taken to produce the first secondary infection (bar chart), both sorted by scenario in primary school setting. (**d**) Box plots showing the number of missed student-days due to classroom closures in the primary school sparked by outbreak. Red text boxes: the first line gives the mean and standard deviation of the total number of student-days missed’ the second line gives the median number.

The primary school setting shows the same cascade of intensifying outbreaks and rapidly mounting student-days of closure as class sizes increase (Fig. 5). This effect occurs in both childcare centres and primary schools because firstly, in a larger classroom it is more likely that a student tests positive for COVID-19. Secondly, when the classroom closes as a result, more students are affected by the closure. Thirdly, because COVID-19 is characterized by presymptomatic infection and aerosol dispersal, there is more infection in larger classrooms before the closure is enacted. Introducing more children into the classroom increases the effective reproductive ratio ($R_e$) for both low and high rates of transmission while cohorting/alternation has little effect (Fig. 5a), and similar scenarios (that is, differing by only 1 educator per class or by weekly alternation) give similar effective reproduction numbers $R_e$ (relative to Fig. 2a). Similar scenarios also give similar out break characteristics, such as the times taken produce the first secondary infection (bar chart) and the number of trials without secondary spread (line) in Fig. 5a.

There is little difference between numbers of missed student-days between the similar scenarios 8:1 and 8(A):1, as well as as 15:1 and 15(A):1 (Fig. 5d). Since the shutdown of a classroom affects both cohorts, there will be very little difference in virus spread between scenarios allotting the same number of students per classroom; this effect is also seen in Fig. 5a. Comparison of Figs. 5a and 3b show similar distributions of outbreak size for all student-teacher ratios, signifying that cohorting does not significantly change the results of structured interactions featured in the model. The true benefit of cohorting arises in the consideration of class sizes, given the desire for contact time with all enrolled students. Comparison of Figs. 4b and 5d shows that the similar scenarios 15:2 RA, 15:1 RA and 15(A):1 RA all result in a comparable number of missed student-days in both low and high transmission scenarios, as do the scenarios 8:2 RA, 8:1 RA and 8(A):1 RA.

Higher student-educator ratios facilitate faster disease spread through the school more than smaller ones (Supplementary Fig. S2). One major difference is the weekly fluctuation of the curves visible in the cohorted scenarios 8(A):1 and 15(A):1. These fluctuations correspond to the rotation of the student cohorts through the school term. Transitions between majority susceptible and recovered regimes is delayed (high transmission) or prevented (low transmission) by cohorting; we see that alternating strategies result in better aggregate infection outcomes, even when classroom capacity is held constant. Scenario 15(A):1 also results in shorter mean and median outbreak lengths in the entire population in both low and high transmission cases (Supplementary Fig S2).

### Sensitivity analyses

We conduct three analyses: in the first, we evaluate the impact of gradual increase of class sizes (by increasing both the number of teachers and children) in both the childcare centre and primary school settings. Secondly, we introduce a reduced time scenario to the childcare centre setting, representing a case where the school-day is shortened to decrease child–child contact in the classroom. Finally, we investigate the effect of varying other model parameters.

#### Unit increases in class size

Our examinations of both the childcare centre and primary school settings featured scenarios in which class sizes varied widely; here we isolate the effect of adding a single child or educator/teacher to each classroom in the institution. Supplementary Fig. S3 shows the effect of adding either a single student (dark blue) or educator/teacher (light blue) to each classroom in the childcare centre. In all panels of Supplementary Fig. S3, adding a single educator to each classroom in the childcare centre makes negligible difference to each of the four measurements presented. Supplementary Fig. S3 shows that the effective reproduction number $R_e$ increases when a child is added to the classroom in the centre, while adding an educator to each room doesn’t significantly increase $R_e$. Similarly, Supplementary Fig. S3 shows that the number of infections increases as a child is added to each room, with almost no change upon the addition of an educator. Similar results are seen in outbreak length (Supplementary Fig. S3) and the number of student-days missed per student (Supplementary Fig. S3); in all scenarios, increases are seen upon the addition of a child to the classroom, with the addition of an educator having very little impact. One can also see that, in each panel, the effects of the addition of a single student to the classroom are exaggerated when students are randomly assigned to classes (RA) as to when siblings are placed together (ST).

We conduct a similar analysis in the primary school setting, where all classrooms have a single teacher and all classroom assignments in the school Bare random. In both low and high transmission scenarios, students can either attend class for all available school days (‘Constant Attendance’), or they can be organised into weekly-rotating cohorts (‘Alternating Cohorts’). In Supplementary Fig. S4, progressively increasing class sizes increases the effective reproduction number ($R_e$, Supplementary Fig. S4), total number of infections in the classrooms (Supplementary Fig. S4), general length of the outbreak in the general populace (Supplementary Fig S4) and the mean number of student-days missed per student (Supplementary Fig. S4). For cases of both constant attendance and alternating cohorts, per-student increase in the number of classroom infections ($R_e$, Supplementary Fig. S4) was greatly enhanced in the high transmission case, due to the multiplicative effect of the new child–child interaction produced by introducing one more student to the classroom.

#### Reduced hours scenario

Given the dependence of infection transmission probability on factors such as the rates of contact between children (and adults), family clustering, classroom size and class time, one potential avenue of risk reduction in both the childcare centre and primary school settings is the reduction of the time that students spend in class. Eq. 3 shows that child–child interaction carries the highest contact rate and Fig. 3b shows that the classroom is the largest site of exposure. In a reduced class time scenario, classroom contact would decline in favour of household contacts. We model the effect of such a reduction in child–child interaction in the primary school setting by halving all classroom and common area infection rates ($\frac{1}{2}\beta ^C$ and $\frac{1}{2}\beta ^O$, respectively), and doubling the household transmission rate ($2\beta ^H$). Reduced-time scenarios are denoted by (B), so that “5(B):2 ST” represents a scenario with 5 students and 2 educators per class, with reduced class time and siblings roomed together. This analysis was not repeated for the childcare centre setting because of the necessity of childcare centres to remain open and provide flexible hours for parents with various daily schedules.

Supplementary Fig. S6 shows that reduced class time “(B)” scenarios are very slightly more conducive to outbreaks (as compared to the other scenarios with a full school-day). However, Supplementary Fig. S5 shows that the reduced-time scenario lowers the effective reproduction number ($R_e$) in the general population (Supplementary Fig. S6) and also decreased the number of infections in the classroom (Supplementary Fig. S5). In both these cases, the reduced-time strategy outperforms weekly alternation strategies. Supplementary Fig. S6 shows that both the reduced-time and weekly alternation strategies result in roughly identical increases in the length of the outbreak in the general population, and both give higher mean numbers of missed student-days per student (Supplementary Fig. S6). Note that the lack of a decline in missed student-days is artificial—students are still losing class time, but by design, rather than due to class shutdown. It appears that way because time is measured in days; one can wonder whether the class time lost to classroom shutdowns would truly outweigh the time lost due to this plan, but that requires using hourly timesteps instead of daily timesteps in our computational model.

#### Varying model parameters

We conducted a sensitivity analysis on $\beta ^H$, $\beta ^C$, $\lambda$ and $R_{init}$ (see Supplementary Information for details). We found that variation in rates of household and classroom interaction and infection ($\beta ^H$ and $\beta ^C$) and the number of individuals initially recovered ($R_{init}$) greatly impact SARS-CoV-2 transmission, but did not change the relative performances of the 22 scenarios. The greatest influence on outcomes remain the scheme of assignment of students to classrooms (RA or ST), the number of students per class (15, 8 or 7), and whether the transmission rate in the classrooms is low or high ($\alpha _C$). Other important factors include classroom closure upon identification of a symptomatic case and the interaction patterns of asymptomatic infected individuals in the household upon classroom closure (i.e. whether they continue to interact in close contact, as would be necessary for younger children, or whether children are old enough to effectively self-isolate). Our baseline assumption was to assume asymptomatic infected individuals who are sent home due to closure of a classroom are able to self-isolate. This assumption is conservative, since inability to self-isolate under these circumstances would result in higher projected outbreak sizes.

## Discussion

We developed and simulated an agent-based model of SARS-CoV-2 transmission in childcare centre and primary school settings for the purposes of informing reopening policies. The model was configured to capture SARS-CoV-2 transmission in a local school building, since many childcare centres operate across several classrooms within schools. These services are an essential bridge for many parents who are unable to drop-off or pick-up children around school hours due to work. Our findings suggest that variability in class size (i.e., number of children in a class) and class composition (i.e., sibling groupings versus random assignment) influence the nature of SARS-CoV-2 transmission within the childcare context. Specifically, a 7:3 student-to-educator ratio that utilized sibling groupings yielded the lowest rates of transmission, while a 15:2 ratio consistently performed far worse. Findings for the primary school ratios show a similar acceleration of negative impacts with increasing class size. Findings from our simulations are sobering, as educators in the province lobbied for a 15 student cap on classrooms in Summer 2020. Our study suggests that classes of this size pose a tangible risk for COVID-19 outbreaks, and that lower ratios would better offset infection and school closures. While school reopening guidelines^10^, public health agencies^31^, and public petitions^32^ have called for smaller class sizes, governments appear to be following some recommendations in reopening plans while not following others.

This accelerating effect of increasing classroom sizes occurs because of three factors working in concert. Firstly, a larger class means that a student is more likely to test positive for COVID-19 at some point. Secondly, when a larger class is closed as a result, it affects more students. Third, presymptomatic transmission and higher densities of students ensure that more children become infected before classroom closure is enacted, resulting in larger outbreak sizes due to more cases both before the closure, and after the closure as the infection continues to spread in households. This particular mechanism is specific to institutional outbreaks for infectious diseases with pre-symptomatic transmission worsened by aerosol transmission routes^25^.

Policies related to childcare and traditional school reopening have not been well integrated^33^. In Ontario, childcare classrooms were capped at a maximum of 10 occupants, overall (hence the 8:2 and 7:3 ratios in the present study)^11^. Conversely, procedures for traditional “school” classrooms have been given the go-ahead for 15 children (hence the 15:2 ratio). While allowable class sizes will differ somewhat as a function of child age and school location, it seems likely that early childhood and elementary school classes may actually surpass these numbers in Ontario. Our findings demonstrate that the 15:2 ratio represents a significantly higher risk, not only for SARS-CoV-2 spread, but for school closures. In one scenario (15:2 random assignment), the modeled outbreak lasted for 105 days. Given that childcare and schools are often operating within the same physical location, this policy discrepancy is questionable. Based on our simulations, a lower ratio (7:3) is indicated. Moreover, it appears that this configuration could be enhanced through the utilization of sibling groupings.

An examination of student-days missed due to classroom closure further elucidates the favourability of smaller class size and sibling grouping as a preventative measure; again, this was observed in both high transmission and low transmission environments. In the most unfavorable scenario (15:2 RA), there were cumulatively 539 and 324 student-days missed in high versus low transmission settings, respectively. Conversely, in the best scenario (7:3, siblings together), there were only 62 and 51 student-days missed. Thus, our simulations suggest that the lower ratios and sibling groupings offer a safeguard against high disruptive classroom closures^34,35^. Given this, a proactive and preventative approach that builds in realistic levels of reduced class time would be better than a reactive strategy that yields unpredictable closure events due to outbreaks.

Several policy and procedural recommendations have emerged from this modeling exercise. First, it is recommended that childcare and school settings, alike, consider lowering student-to-educator ratios. Commensurate with the present findings, a 7:3 ratio (10 individuals per class including both children and adults) outperforms a 15:2 ratio on key metrics. Second, there also appears to be benefit associated with sibling groupings. Thus, a siblings together configuration should be considered. Third, the majority of transmission occurred in the classroom. As such, it is important for reopening plans to consider social distancing and hygiene procedures within classrooms - a recommendation that may only be feasible with fewer children in the classroom. It is unlikely that classrooms with 15 or more children will afford children with the necessary space to socially distance. Finally, in the primary school settings, significant benefits accrue for 15(A):1 relative to the 30:1 student-educator ratio, and thus decision-makers should reconsider the conventional model of putting 30 students in classrooms every day in favour of cohorts of 15 students alternating weekly. Implementing half-days in schools could also be considered.

These results are also relevant for authorities facing the possibility of re-closing schools in light of an impending resurgence of COVID-19 due to a second or third pandemic wave. For instance, a combination of tamping down community transmission, in combination with restructuring schools according to some of these strategies (such as implementing half days or alternating cohorts) could enable schools to remain partially open during the resurgence, instead of needing to close them altogether^36^.

The impact of school closure on the transmission of COVID-19 is a widely studied topic^37^. However, multiple sources of bias must be considered, for instance on account of higher rates of asymptomatic transmission in children^38^. Due to asymptomatic transmission, outbreaks in schools may be harder to identify, and public health may classify adults in associated households as the index case of an outbreak, instead of asymptomatic children who brought the infection into the home. A systematic review of studies of the impact of school closure on COVID-19 transmission found that most studies identify an effect of closures, although a smaller number of studies less subject to biases find no effect^39^. Empirical validation of our model would entail analysis of outbreak sizes as they depend upon classroom size. This would require both accurate data on school outbreak sizes as well as knowing the class sizes in the affected school districts. This could be studied in future research.

Finally, the present study has a number of limitations that should be considered. While it is becoming increasingly clear that COVID-19 risk varies as a function of social determinants of health (e.g., socioeconomic status, race, ethnicity, immigration status, neighborhood risk), along with opportunities for social distancing^40^, the present study did not take these considerations into account. This present study also didn’t directly consider any difference in compliance between children of difference ages, though our consideration of low and high transmission scenarios represent differing levels of compliance with health regulations in both childcare centre and primary school contexts. Future simulation studies might consider how these social determinants intersect with childcare and school configurations. Also, the measures presented - decreasing class sizes, dividing large classes into smaller cohort alternating between in-class and online instruction, and limiting schooling to half-days - could be subjected to a cost-benefit analysis, where their effects on disease spread are weighed against any requisite impacts on learning, healthcare expenditure and the emotional well-being of students, teachers and parents. Additionally, we have not considered the effects of COVID-19 testing, since asymptomatic individuals are not prioritised in testing programmes, and many current health and safety policies simply ask whether the individual is experiencing any symptoms of COVID-19, upon which they are told to self-isolate. In the present model, classrooms are closed upon detection of a symptomatic case. This is representative of current reopening plans, so the effects of random COVID-19 testing of asymptomatic individuals was not treated here. Finally this study was primarily concerned with SARS-CoV-2 infection and student-days lost. That being said, there are many important outcomes to consider in relation to children’s developmental health in the pandemic. Longitudinal studies considering children’s learning and mental health outcomes in relation to new childcare and school configurations are strongly indicated^41^.

## Materials and methods

### Population structure

There are *N* households in the population, and a single educational institution (either a school or a school, dependent on scenarios to be introduced later) with *M* rooms and a maximum capacity dependent on the scenario being tested. Effective contacts between individuals occur within each household, as well as rooms and common areas (entrances, bathrooms, hallways, etc.) of the institution. All groups of individuals (households and rooms) in the model are assumed to be well-mixed.

Each individual (agent) in the model is assigned an age, household, room in the childcare facility and an epidemiological status. Age is categorical, so that every individual is either considered a child (C) or an adult (A). Epidemiological status is divided into stages in the progression of the disease; agents can either be susceptible (*S*), exposed to the disease (*E*), presymptomatic (an initial asymptomatic infections period *P*), symptomatically infected (*I*), asymptomatically infected (*A*) or removed/recovered (*R*), as shown in Fig. 1b.

In the model, some children in the population are enrolled as students in the institution and assigned a classroom based on assumed scenarios of classroom occupancy while some adults are assigned educator/caretaker roles in these classroom (again dependent on the occupancy scenario being tested). Assignments are made such that there is only one educator per household and that children do not attend the same institution as a educator in the household (if there is one), and *vice versa*.

### Interaction and disease progression

The basic unit of time of the model is a single day, over which each attendee (of the institution) spends time at both home and at the institution. The first interactions of each day are established within each household, where all members of the household interact with each other. An asymptomatically infectious individual of age *i* will transmit the disease to a susceptible housemate with the age *j* with probability $\beta ^H_{i,j}$, while symptomatically infectious members will self-isolate (not interact with housemates) for a period of 14 days.

The second set of interpersonal interactions occur within the institution. Individuals (both students and educators) in each room interact with each other, where an infectious individual of age *i* transmits the disease to some susceptible individual of age *j* with probability $\beta ^C_{i,j}$. To signify common areas within the building (such as hallways, bathrooms and entrances), each individual will then interact with every other individual in the institution. There, an infectious individual of age *j* will infect a susceptible individual of age *i* with probability $\beta ^O_{i,j}$.

To simulate community transmission (for example, public transport, coffee shops and other sources of infection not explicitly modelled here), each susceptible attendee is infected with probability $\lambda _S$. Susceptible individuals not attending the institution in some capacity are infected at rate $\lambda _N$, where $\lambda _N>\lambda _S$ to compensate for those consistent effective interactions outside of the institution that are neglected by the model (such as workplace interactions among essential workers and members of the public).

Figure 1b shows the progression of the illness experienced by each individual in the model. In each day, susceptible (*S*) individuals exposed to the disease via community spread or interaction with infectious individuals (those with disease statuses *P*, *A* and *I*) become exposed (*E*), while previously exposed agents become presymptomatic (*P*) with probability $\delta$. Presymptomatic agents develop an infection in each day with probability $\delta$, where they can either become symptomatically infected (*I*) with probability $\eta$ or asymptomatically infected (*A*) with probability $1-\eta$.

The capacity of the sole educational institution in the model is divided evenly between 5 rooms, with class size and student-educator ratio governed by one of three basic scenarios: seven students and three educators per room (7 : 3), eight students and two educators per room (8 : 2), and fifteen students and two educators per room (15 : 2). Classroom assignments for children can be either randomized or grouped by household (siblings are put in the same class).

Symptomatically infected agents (*I*) are removed from the simulation after 1 day (status *R*) with probability $\gamma _I$, upon which they self-isolate for 14 days, and therefore no longer pose a risk to susceptible individuals. Asymptomatically infected agents (*A*) remain infectious but are presumed able to maintain regular effective contact with other individuals in the population due to their lack of noticeable symptoms; they recover during this period (status *R*) with probability $\gamma _A$. Disease statuses are updated at the end of each day, after which the cycles of interaction and infection reoccur the next day.

The actions of symptomatic (status *I*) agents depend on age and role. Individuals that become symptomatic maintain a regular schedule for 1 day following initial infection (including effective interaction within the institution, if attending), after which they serve a mandatory 14-day isolation period at home during which they interaction with no one (including other members of their household). On the second day after the individual’s development of symptoms, their infection is considered a disease outbreak centred in their assigned room, triggering the closure of that room for 14 days. All individuals assigned to that room are sent home, where they self-isolate for 14 days due to presumed exposure to the disease. Symptomatically infected children are not replaced, and simply return to their assigned classroom upon recovery. At the time of classroom reopening, any symptomatic educator is replaced by a substitute for the duration of their recovery, upon which they reprise their previous role in the institution; the selection of a substitute is made under previous constraints on educator selection (one educator per household. with no one chosen from households hosting any children currently enrolled in the institution).

### Parameterisation

The parameter values are given in Supplementary Table S4. The sizes of households in the simulation was determined from 2016 Statistics Canada census data on the distribution of family sizes^42^. We note that Statistics Canada data only report family sizes of 1, 2 or 3 children: the relative proportions for 3+ children were obtained by assuming that $65 \%$ of families of 3+ children had 3 children, $25\%$ had 4 children, $10\%$ had 5 children, and none had more than 5 children. Each educator was assumed to be a member of a household that did not have children attending the school. Again using census data, we assumed that $36\%$ of educators live in homes with no children, where an individual lives alone with probability 0.282, while households hosting 3, 4, 5, 6, and seven adults occur with probability 0.345, 0.152, 0.138, 0.055, 0.021 and 0.009 respectively. Others live with $\ge 1$ children in households following the size and composition distribution depending on the number of adults in the household. For single-parent households, a household with a single child occurs with probability 0.169, and households with 2, 3, 4 and 5 children occur with probabilities 0.079, 0.019, 0.007 and 0.003 respectively. With two-parent households, those probabilities become 0.284, 0.307, 0.086, 0.033 and 0.012.

The age-specific transmission rates in households are given by the matrix:

1 $$\begin{aligned} \begin{bmatrix} \beta ^H_{1,1} &{} \beta ^H_{1,2} \\ \beta ^H_{2,1} &{} \beta ^H_{2,2} \\ \end{bmatrix} \equiv \beta ^H \begin{bmatrix} c^H_{1,1} &{} c^H_{1,2} \\ c^H_{2,1} &{} c^H_{2,2} \\ \end{bmatrix}, \end{aligned}$$

where $c^H_{i,j}$ gives the number of contacts per day reported between individuals of ages *i* and *j* estimated from data^28^ and the baseline transmission rate $\beta ^H$ is calibrated. To estimate $c^H_{i,j}$ from the data in Ref.^28^, we used the non-physical contacts of age class 0–9 years and 25–44 years of age with themselves and one another in Canadian households. Based on a meta-analysis, the secondary attack rate of SARS-CoV-2 appears to be approximately $15 \%$ on average in both Asian and Western households^43^. Hence, we calibrated $\beta ^H$ such that a given susceptible person had a $15 \%$ chance of being infected by a single infected person in their own household over the duration of their infection averaged across all scenarios tested. As such, age specific transmission is given by the matrix

2 $$\begin{aligned} \beta ^H\cdot \begin{bmatrix} 0.5378 &{} 0.3916 \\ 0.3632 &{} 0.3335 \end{bmatrix}. \end{aligned}$$

To determine $\lambda _S$ we used case notification data from Ontario during lockdown, when schools, workplaces, and schools were closed^44^. During this period, Ontario reported approximately 200 cases per day. The Ontario population size is 14.6 million, so this corresponds to a daily infection probability of $1.37 \times 10^{-5}$ per person. However, cases are under-ascertained by a significant factor in many countries. We assumed an under-ascertainment factor of 8.45 based on an empirical estimate of under-reporting^45^, meaning there are actually 8.45 times more cases than reported in Ontario, giving rise to $\lambda _S = 1.16 \times 10^{-4}$ per day; $\lambda _N$ was set to $2\cdot \lambda _S$. We emphasize that this number may fall later in the pandemic as testing capacity increases, although some individuals may still never get tested–especially schoolchildren, who are often asymptomatic.

The age-specific transmission rates in the school rooms is given by the matrix

3 $$\begin{aligned} \begin{bmatrix} \beta ^C_{1,1} &{} \beta ^C_{1,2} \\ \beta ^C_{2,1} &{} \beta ^C_{2,2} \\ \end{bmatrix} \equiv \beta ^C \begin{bmatrix} c^C_{1,1} &{} c^C_{1,2} \\ c^C_{2,1} &{} c^C_{2,2} \\ \end{bmatrix} \equiv \beta ^C \begin{bmatrix} 1.2356 &{} 0.0588 \\ 0.1176 &{} 0.0451 \end{bmatrix}, \end{aligned}$$

where $c^C_{i,j}$ is the number of contacts per day reported between age *i* and *j* estimated from data^28^. To estimate $c^C_{i,j}$ from the data in Ref.^28^, we used the non-physical contacts of age class 0–9 years and 20–54 years of age, with themselves and one another, in Canadian schools. Epidemiological data on secondary attack rates in educational institutions are rare, since childcare centres and schools were closed early in the outbreak in most areas. We note that contacts in families are qualitatively similar in nature and duration to contacts in schools with small group sizes, although these contacts are generally more dispersed among the larger groups in rooms than among the smaller groups in households. On the other hand, rooms may represent equally favourable conditions for aerosol transmission, as opposed to close contact. Hence, we assumed that $\beta ^C = \alpha _C \beta ^H$, with a baseline value of $\alpha _C = 0.75$ based on more dispersed contacts expected in the larger room group, although we varied this assumption in sensitivity analysis.

To determine $\beta ^O$ we assumed that $\beta ^O = \alpha _O \beta ^C$ where $\alpha _O \ll 1$ to account for the fact that students spend less time in common areas than in their rooms. To estimate $\alpha _O$, we note that $\beta ^O$ is the probability that a given infected person transmits the infection to a given susceptible person. If students and staff have a probability *p* per hour of visiting a common area, then their chance of meeting a given other student/staff in the same area in that area is $p^2$. We assumed that $p=0.05$ and thus $\alpha _O = 0.0025$. The age-specific contact matrix for $\beta ^O$ was the same as that used for $\beta ^C$ (Eq. 3).

### Model initialization

Upon population generation, each agent is initially susceptible (*S*). Individuals are assigned to households as described in the “Parameterisation” section, and children are assigned to rooms either randomly or by household. We assume that parents in households with more than one child will decide to enroll their children in the same institution for convenience with probability $\xi =80\%$, so that each additional child in multi-child households will have probability $1-\xi$ of not being assigned to the institution being modelled.

Households hosting educators are generated separately. As in the “Parameterisation” section, we assume that $36\%$ of educators live in adult-only houses, while the other educators live in houses with children, both household sizes following the distributions outlined in the “Parameterisation” section. The number of educator households is twice that required to fully supply the school due to the replacement process for symptomatic educators outlined in the “Disease Progression” section.

Initially, a proportion of all susceptible agents $R_{init}$ is marked as removed/recovered (*R*) to account for immunity caused by previous infection moving through the population. A single randomly chosen school attendee is chosen as a primary case and is made presymptomatic (*P*) to introduce a source of infection to the model. All simulations are run until there are no more potentially infectious (*E*, *P*, *I*, *A*) individuals left in the population and the institution is at full capacity. All results were averaged over 2000 trials.

### Estimating * β*^H^

Agents in the simulation were divided into two classes: “children” (ages 0–9) and “adults” (ages 25–44). Available data on contact rates^28^ was stratified into age categories of width 5 years starting at age 0 (0–5, 5–9, 10–14, etc.). The mean number of contacts per day $c_{i,j}^H$ for each class we considered (shown in Eq. 2) was estimated by taking the mean of the contact rates of all age classes fitting within our presumed age ranges for children and adults.

For $\beta ^H$ calibration, we created populations by generating a sufficient number of households to fill the institution in each of the three tested scenarios; 15 : 2, 8 : 2 and 7 : 3. In each household, a single randomly chosen individual was infected (each member with equal probability) by assigning them a presymptomatic disease status *P*; all other members were marked as susceptible (disease status *S*). In each day of the simulation, each member of each household was allowed to interact with the infected member, becoming exposed to the disease with probability given in Eq. 2. Upon exposure, they were assigned disease status *E*. At the beginning of each subsequent day, presymptomatic individuals proceeded to infected statuses *I* and *A*, and infected agents were allowed to recover as dictated by Fig. 1b and Supplementary Table S4. This cycle of interaction and recovery within each household was allowed to continue until all infected individuals were recovered from illness.

We did not allow exposed agents (status *E*) to progress to an infectious stage (*I* or *A*) since we were interested in finding out how many infections within the household would result *from a single infected household member*, as opposed to added secondary infections in later days. At the end of each trial, the specific probability of infection ($\pi _n$) in each household $H_n$ was calculated by dividing the number of exposed agents in the household ($E_n$) by the size of the household $|H_n|$ less 1 (accounting for the member initially infected). Single occupant households ($|H_n|=1$) were excluded from the calculation. The total probability of infection $\pi$ was then taken as the mean of all $\pi _n$, so that

4 $$\begin{aligned} \pi =\frac{1}{D}\sum _{n}\pi _n=\frac{1}{D}\sum _{|H_n|\ge 2}\frac{E_n}{|H_n|-1}, \end{aligned}$$

where *D* represents the total number of multiple occupancy households in the simulation. This modified disease simulation was run for 2000 trials each of different prospective values of $\beta ^H$ ranging from 0 to 0.21. The means of all corresponding final estimates of the infection rate were taken per value of $\beta ^H$, and the value corresponding to a infection rate of $15\%$ was interpolated.

### Simplifying assumptions

Our model makes simplifying assumptions that may influence its predictions. For instance, we assume that classrooms are homogeneously mixing and did not take social structure into account. Social structure might slow the spread of COVID-19 in classrooms. We also assumed that public health authorities will respond to a confirmed case by closing the classroom, although in practice, they may keep the class running if they think the case does not represent an infection risk to children or adults. This would reduce the number of student-days lost to closure. Similarly, we did not account for potential contacts between school children outside of classes, although students of a classroom that has been closed may still interact with their classmates outside of school. Other simplifying assumptions are mentioned in the “Discussion” section.

## Supplementary Information

Supplementary Information.

## Data Availability

The datasets generated during and/or analysed during the current study are available from the corresponding author on reasonable request.
