# Supplementary material for: Model-based projections for COVID-19 outbreak size and student-days lost to closure in Ontario childcare centres and primary schools
Source: Sci Rep. 2021 Mar 18;11:6402. doi: 10.1038/s41598-021-85302-6 (PMC7973423; doi:10.1038/s41598-021-85302-6)
Supplement: Supplementary file 1 — Supplementary Information. [file 41598_2021_85302_MOESM1_ESM.pdf]

Model-based projections for COVID-19 outbreak size and student-days lost to closure in Ontario childcare centres and primary schools

SUPPLEMENTARY INFORMATION

Brendon Phillips
Department of Applied Mathematics, University of Waterloo, Waterloo, Ontario, Canada

Dillon T. Browne
Department of Psychology, University of Waterloo, Waterloo, Ontario, Canada

Madhur Anand
School of Environmental Sciences, University of Guelph, Guelph, Ontario, Canada

Chris T. Bauch
Department of Psychology, University of Waterloo, Ontario, Canada

Tables of the various scenarios considered

The scenarios considered in this study are combinations of factors potentially affecting the speed and efficiency of transition within both childcare centres and primary schools. In Column 1 of Tabs. S1 and S2, the case of 'high' transmission rate encapsulates behaviours facilitating disease spread (such as close contact, insufficient disinfection and ventilation of student spaces, etc), while the 'low' transmission case representing obedience to safety guidelines (such as physical distancing. mask usage, hand disinfection and other such measures). For considering different class sizes and composition, we change the numbers and ratios of students and teachers in each classroom of the centre or school (Column 2 of both Tabs. S1 and S2). Also common to both childcare centre and schools is the variation of the duration of the school day, as shown in Column 4 of both Tabs. S1 and S2. Students can either attend class for some typical duration (full), or can instead spend less time in class to lessen the number of contacts in the institution (reduced, B).

Childcare Centre (no student cohorts)			
**Transmission Rate**	**Student/Teacher Ratio**	**Classroom Assignment**	**Schooling Duration**
High transmission	7 students, 3 teachers (7:3)	Siblings together (ST)	Full (blank)
			Reduced (B)
		Random Assignment (RA)	
			B
	8 students, 2 teachers (8:2)	ST	
			B
		RA	
			B
	15 students, 2 teachers (15:2)	ST	
			B
		RA	
			B
Low transmission	7 students, 3 teachers (7:3)	ST	
			B
		RA	
			B
	8 students, 2 teachers (8:2)	ST	
			B
		RA	
			B
	15 students, 2 teachers (15:2)	ST	
			B
		RA	
			B

Table S1. Scenarios evaluated based on different assumptions about transmission probabilities, educator-student ratios, student assignment to classrooms and the duration of the school day in childcare centres.

Uniquely in childcare centres (in this study), students can be assigned to classrooms either without pattern (random assignment, RA) or by placing cohabiting students together where possible (siblings together, ST); this choice is shown in Column 3 of Tab. S1. Here, we assume that all students and teachers attend the childcare centre whenever possible. However, in the case of primary schools, we treat all classroom assignment of students as random, and instead model scenarios where students are placed in alternating cohorts (A) or allowed to attend class on all school days (no cohorting). This choice appears of Column 3 of Tab. S2.

Sensitivity Analysis: varying α_0 and B_H

The parameter β^H represents the rate of interaction in the household, and thereby regulates the spread of the disease. For each value of α_0, increasing the rate of interaction in the home β^H increases the number of infections produces for both RA (Supplementary Fig. S7) and ST (Supplementary Fig. S8) assignment. In most scenarios (7:3 RA being one of the exceptions), varying α_0 (for constant β^H) produces a small increase in the number of infections produced throughout the simulation. The rate of increase also depends on the number of children in the classroom; for the scenario 31:1 RA, increasing β^H from 0.0545 to its baseline value 0.109 almost triples the number of total infections.

Sensitivity Analysis - Varying α_0 and R_{init}

The parameter R_{init} refers to the proportion of individuals we presume are recovered from some previous period of infection spread, while α_0 is responsible for the rate of infection in common areas relative to the infection rate in the classroom. All other parameters are set to the baseline values given in Supplementary Tab. S4. These parameters were varied together by 50% in

Primary School (all students allocated randomly)			
**Transmission Rate**	**Student/Teacher Ratio**	**Number of Cohorts**	**Schooling Duration**
High transmission	8:1	No cohorting (blank)	Full (blank)
			Reduced (B)
		Two alternating cohorts (A)	
			B
	15:1		
			B
		A	
			B
	30:1		
			B
		A	
			B
Low transmission	8:1		
			B
		A	
			B
	15:1		
			B
		A	
			B
	30:1		
			B
		A	
			B

Table S2. Scenarios evaluated based on different assumptions about transmission probabilities, educator-student ratios, alternating cohorts and duration of the school day in primary schools.

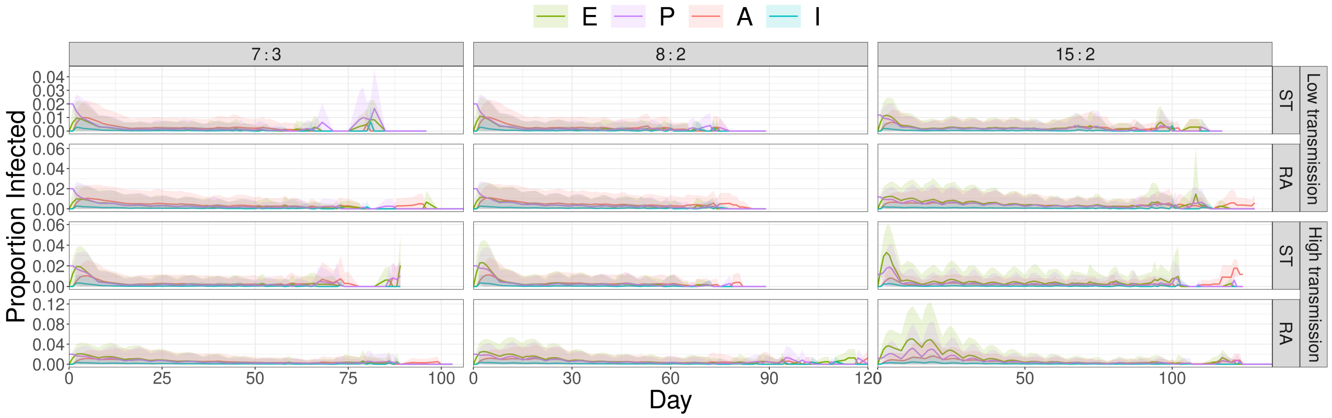

Figure S1. Time series of the proportions of exposed (E), presymptomatic (P), asymptomatic (A) and infected (I) individuals in the simulation for each scenario in the childcare centre setting. The ensemble means are represented by solid lines, while the respected shaded ribbons show one standard deviation of the results.

either direction. In Supplementary Figs. S9 and S10, increasing values of R_{init} lower both the means and standard deviations of the total number of infections for each value of α_0. Also, for each value of R_{init}, the total number of infections produced increases with α_0 . This shows opposing interaction between increasing common area infection and increasing initial recovery rate; one increases infection and the other lowers it (respectively).

Sensitivity Analysis - Varying α_0 and λ_i

From Tab. S4, parameter λ_i varies the amount of community infection in the model (infection due to other sources not modelled, such as public transport); be reminded that we assumed that the rate of community infection is effectively twice the baseline value for those individuals in the model not attending the school.

For each value of α_0 in Supplementary Fig. S12, the total number of infections produced in the simulation increases with λ in each scenario with random assignment (RA), and also with grouping by household (ST, Supplementary Fig. S11). For each

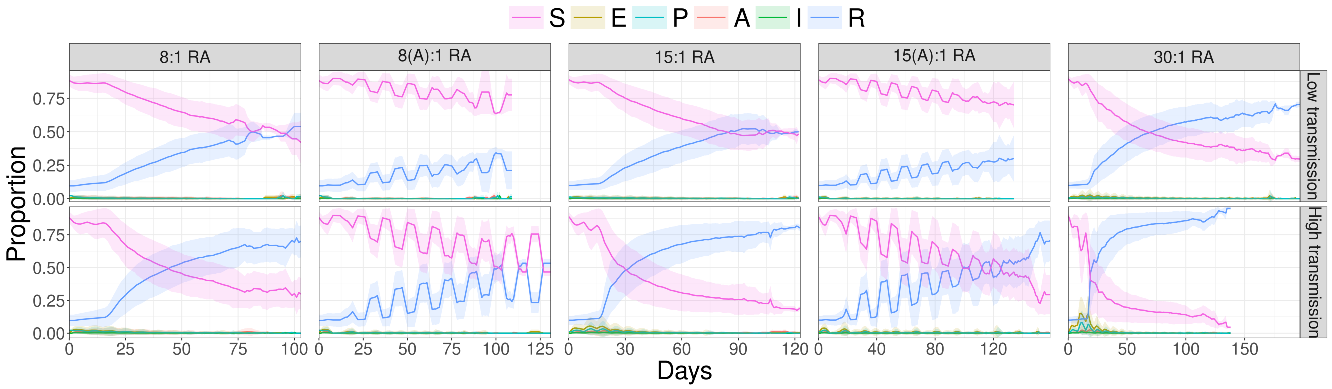

(a) Time series showing the trends in the mean proportions of current primary school attendees in each stage of disease progression. Shaded ribbons around each curve show one standard deviation of the averaged time series.

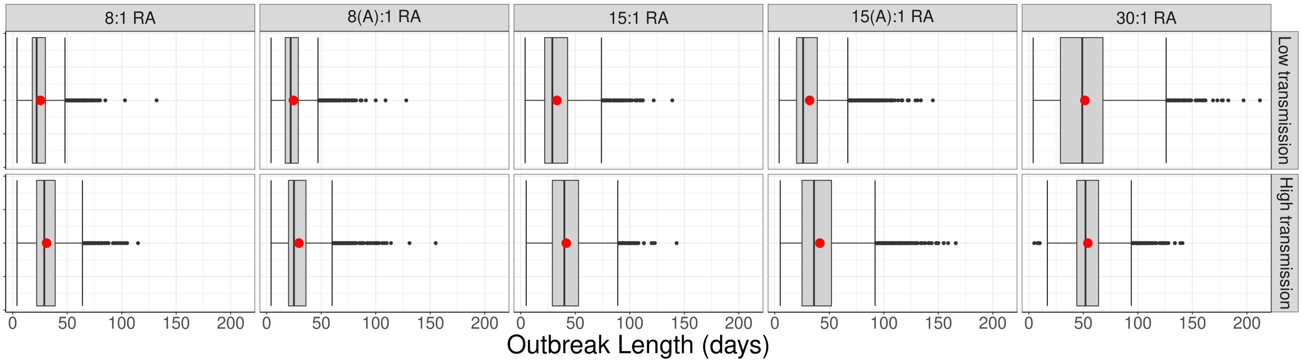

(b) Box plots depicting the distribution of simulation durations for each scenario, describing the length of the outbreak in a primary school setting.

Figure S2. Trends in illness and outbreak length in the primary school setting.

λ, there is no consistent relationship between the numbers of infections and the value of α_0. This result is intuitive; though the effect is not pronounced, increasing the rate of community infection increases the total number of infections in each tested scenario.

Supplementary Figures and Tables

References

1. Nishiura, H., Linton, N. M. & Akhmetzhanov, A. R. Serial interval of novel coronavirus (2019-ncov) infections. *medRxiv* (2020).
2. Tindale, L. *et al.* Transmission interval estimates suggest pre-symptomatic spread of COVID-19. *medRxiv* (2020).
3. Prem, K., Cook, A. R. & Jit, M. Projecting social contact matrices in 152 countries using contact surveys and demographic data. *PLoS computational biology* **13**, e1005697 (2017).
4. Koh, W. C. *et al.* What do we know about sars-cov-2 transmission? a systematic review and meta-analysis of the secondary attack rate, serial interval, and asymptomatic infection. *medRxiv* (2020).
5. Public Health Ontario. Ontario covid-19 data tool. https://www.publichealthontario.ca/en/data-and-analysis/infectious-disease/covid-19-data-surveillance/covid-19-data-tool (accessed June 10, 2020).
6. Canada, S. Statistics canada 2016 census. https://www12.statcan.gc.ca/census-recensement/2016/dp-pd/prof/details/page.cfm (accessed June 9, 2020).

			Peak Time			Maximum $(\times 10^{-4})$		
α_C	**Status**	**assignment**	**15:2**	**8:2**	**7:3**	**15:2**	**8:2**	**7:3**
**0.75**	**P**	**RA**	12	1	1	319	200	200
		**ST**	4	1	1	188	200	200
	**E**	**RA**	12	3	3	503	252	210
		**ST**	3	3	2	330	231	192
	**I**	**RA**	12	2	2	45	35	34
		**ST**	4	2	2	28	35	34
	**A**	**RA**	19	5	5	163	119	116
		**ST**	4	5	5	80	113	104
α_C	**Status**	**assignment**	**15:2**	**8:2**	**7:3**	**15:2**	**8:2**	**7:3**
**0.25**	**P**	**RA**	1	1	1	118	200	200
		**ST**	1	1	1	118	200	200
	**E**	**RA**	5	3	2	123	114	98
		**ST**	3	2	2	117	110	95
	**I**	**RA**	2	2	2	20	27	28
		**ST**	2	2	2	20	29	28
	**A**	**RA**	5	5	5	67	109	105
		**ST**	5	4	4	65	100	99

Table S3. Times at which the mean proportions of presymptomatic (P), exposed (E), symptomatically infected (I) and asymptomatically infected (A) school attendees peak during the first 30 days of simulation with secondary spread with respect to each of the scenarios tested, and the corresponding peak number of cases.

Parameter	Meaning	Baseline Value	Source
η	probability of symptomatic infection	0.6 (adults)	TBD
		0.4 (children)	TBD
δ	transition probability, $E \to P$	0.5/day	[1,2]
σ	transition probability, $P \to I, A$	0.5/day	[1,2]
γ_I	transition probability, $I \to R$	1.0/day	[1,2]
γ_A	transition probability, $A \to R$	0.25/day	[1,2]
c_{ij}^H	household contact matrix	...	[3]
β^H	transmission probability in households	0.109	[4], calibrated
c_{ij}^C	room contact matrix	...	[3]
β^C	transmission probability in classrooms	$\beta^C = \alpha_C \beta^H$,	[4], assumption
		$\alpha_C = 0.75$	
β_{ij}^O	transmission probability in common areas	$\beta^O = \alpha_O \beta^C$,	[3,4], assumption
		$\alpha_O = 0.0025$	
λ_i	infection rate due to other sources	1.16×10^{-4}/day	[5], estimated
R_{init}	initial proportion with immunity	0.1	assumption
ξ	probability of siblings attending same centre	0.8	assumption
o	proportion of childless educators	0.36	[6], assumption
	household size distributions		[6]

Table S4. Parameter definitions, baseline values and literature sources.

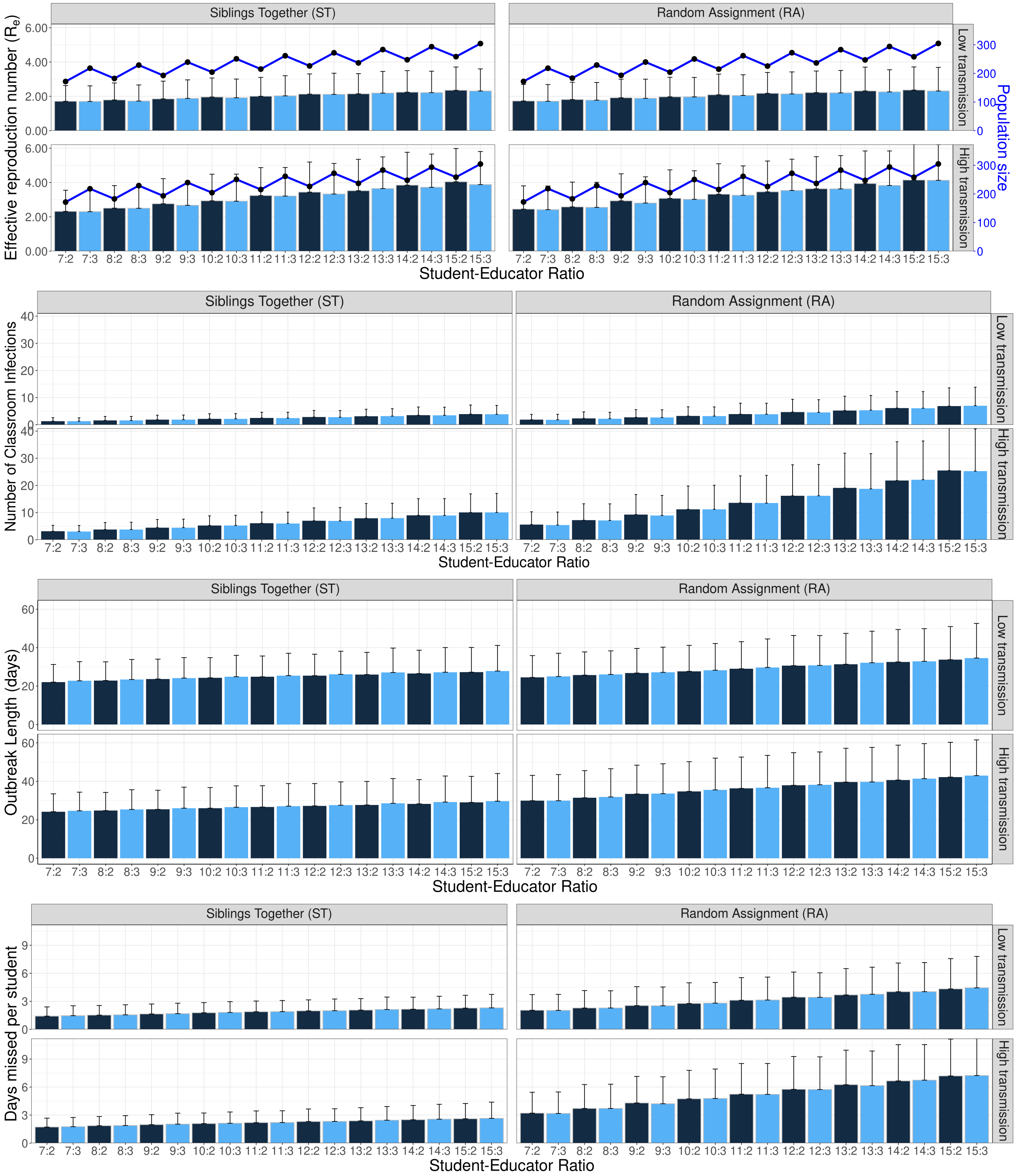

Figure S3. Diagram showing the effects of increasing class size progressively by one unit (either student or educator) in the childcare centre setting. Light blue bars represent scenarios with 2 educators per classroom, while dark blue represents scenarios placing 3 educators in each classroom. Error bars denote one standard deviation. (A) Effective reproductive ratio R_e (bars) and total population size (line). (B) The total number of infections in classrooms in the centre. (C) The length of the outbreak in the entire community (not just among childcare centre attendees). (D) The mean number of student-days missed per student.

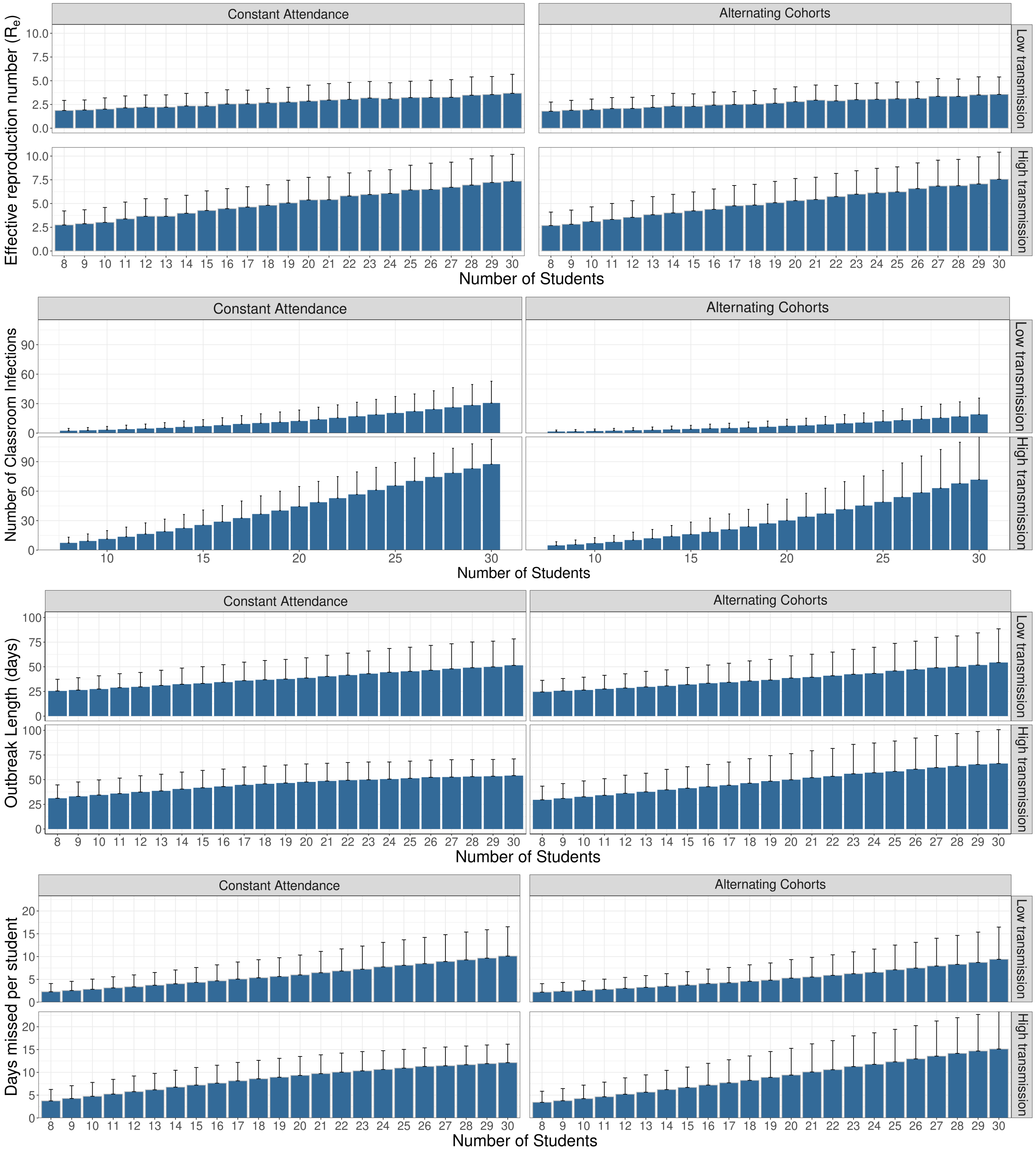

Figure S4. Diagram showing the effects of increasing class size progressively by a single student in the primary school setting (each classroom will have a single teacher, and classroom assignments are done at random). Error bars denote one standard deviation. (A) Effective reproductive ratio R_e. (B) The total number of infections in the classrooms of the centre. (B) The length of the outbreak in the entire community (not just among school attendees). (D) The mean number of student-days missed per student.

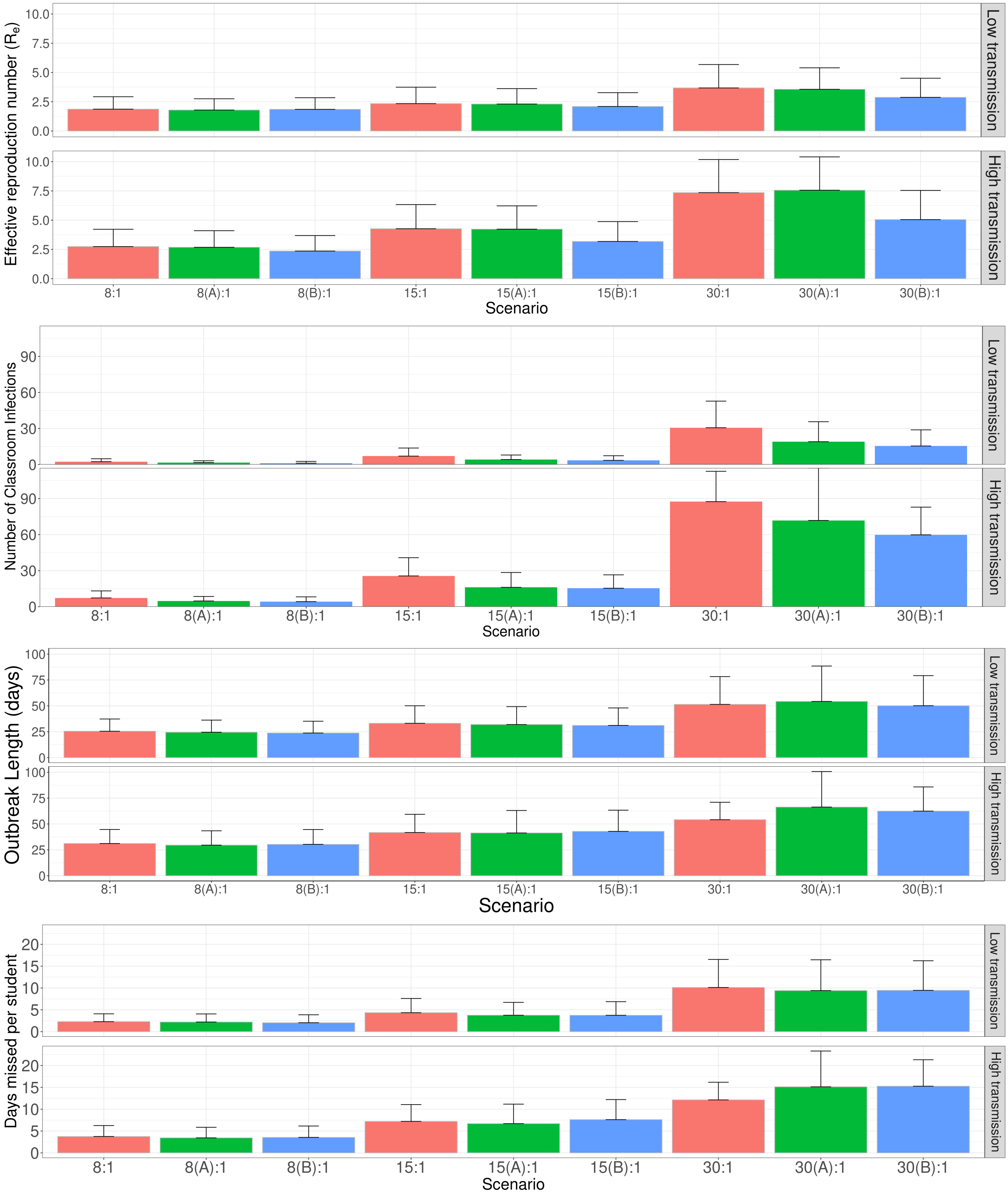

Figure S5. The effect of reduction of the duration of the school day in the primary school setting. (A) Effective reproduction number R_e. (B) The number of infections in the classroom. (C) Length of the initial outbreak in the entire population. (D) The mean number of student-days missed per student. Light blue bars represent reduced-time scenarios, while red and dark blue bars represent full-length school say scenarios.

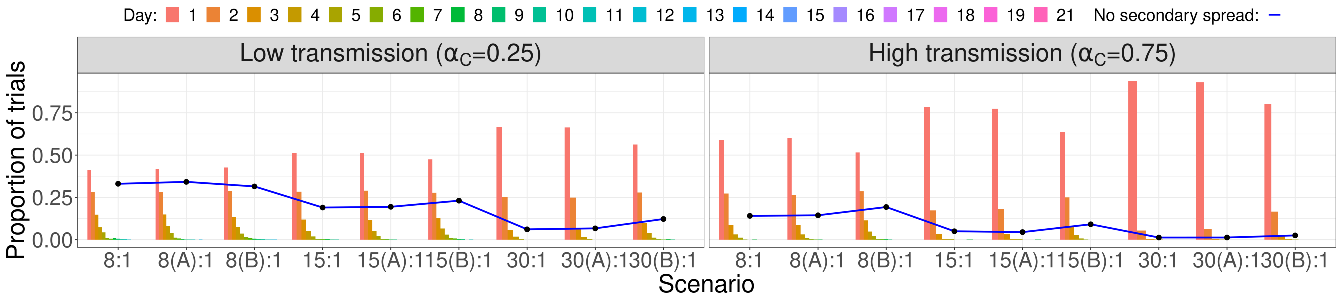

Figure S6. Diagram showing the proportion of trials without secondary spread (curve) and the time taken to produce the first secondary infection (bar chart) in the primary school setting with both alternating weekly cohorts and reduced time strategies.

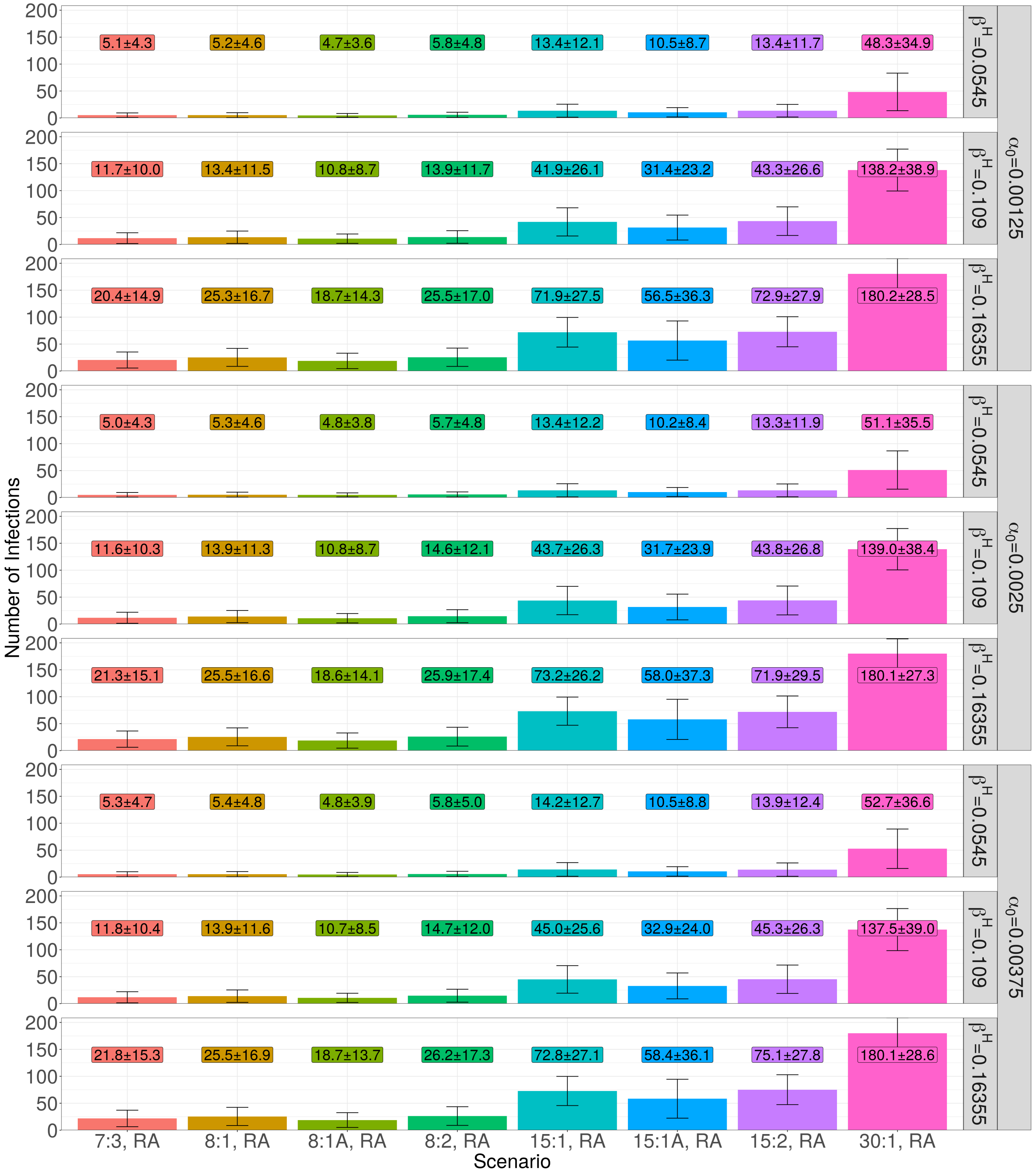

Figure S7. Results of varying the parameters β^H and α_0 by (50% each) on the total number of produced infections for RA assignment. Error bars denote a single standard deviation of the data used, and boxed text shows the corresponding mean and standard deviation.

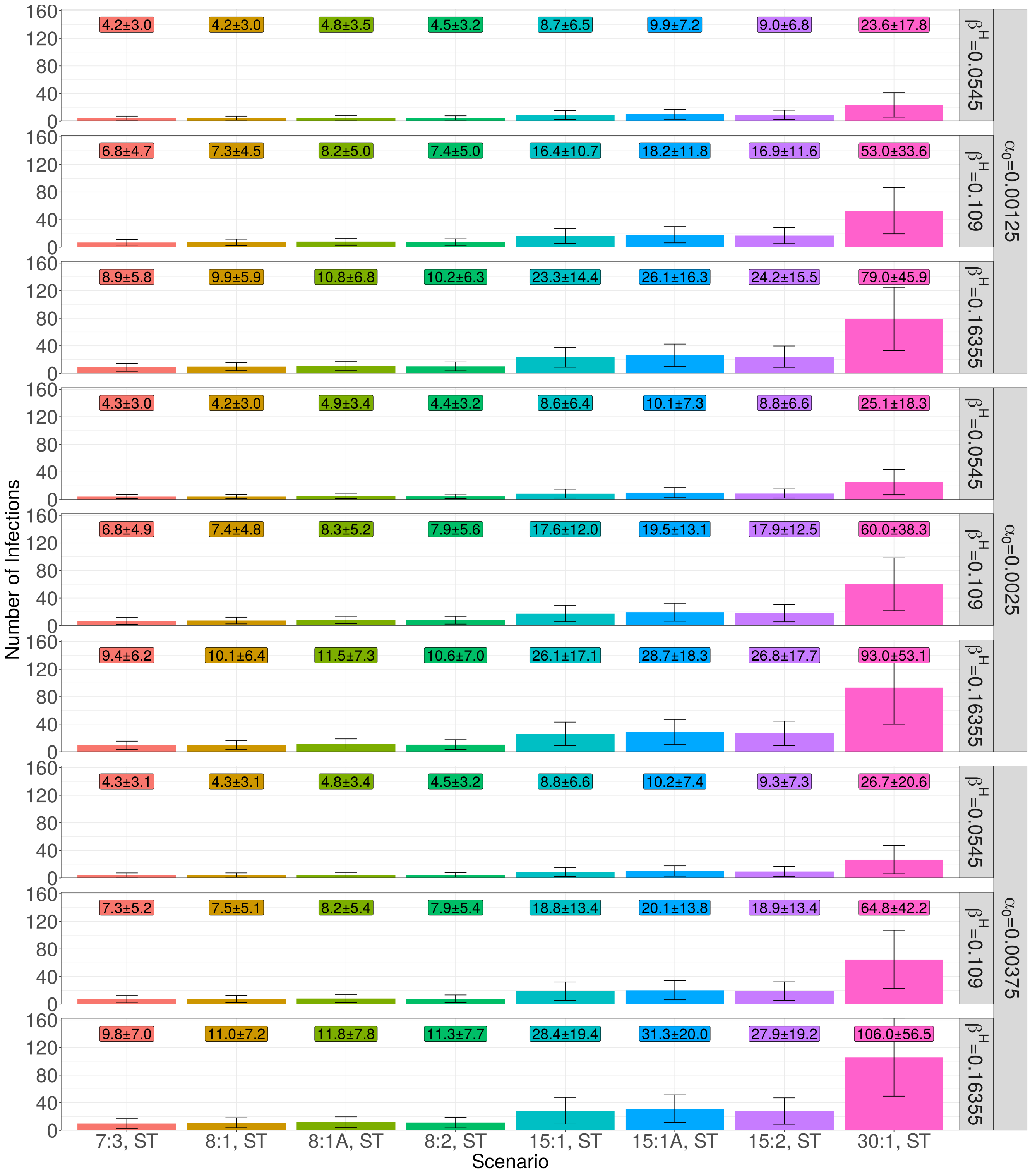

Figure S8. Results of varying the parameters β^H and α_0 by (50% each) on the total number of produced infections for ST assignment. Error bars denote a single standard deviation of the data used, and boxed text shows the corresponding mean and standard deviation.

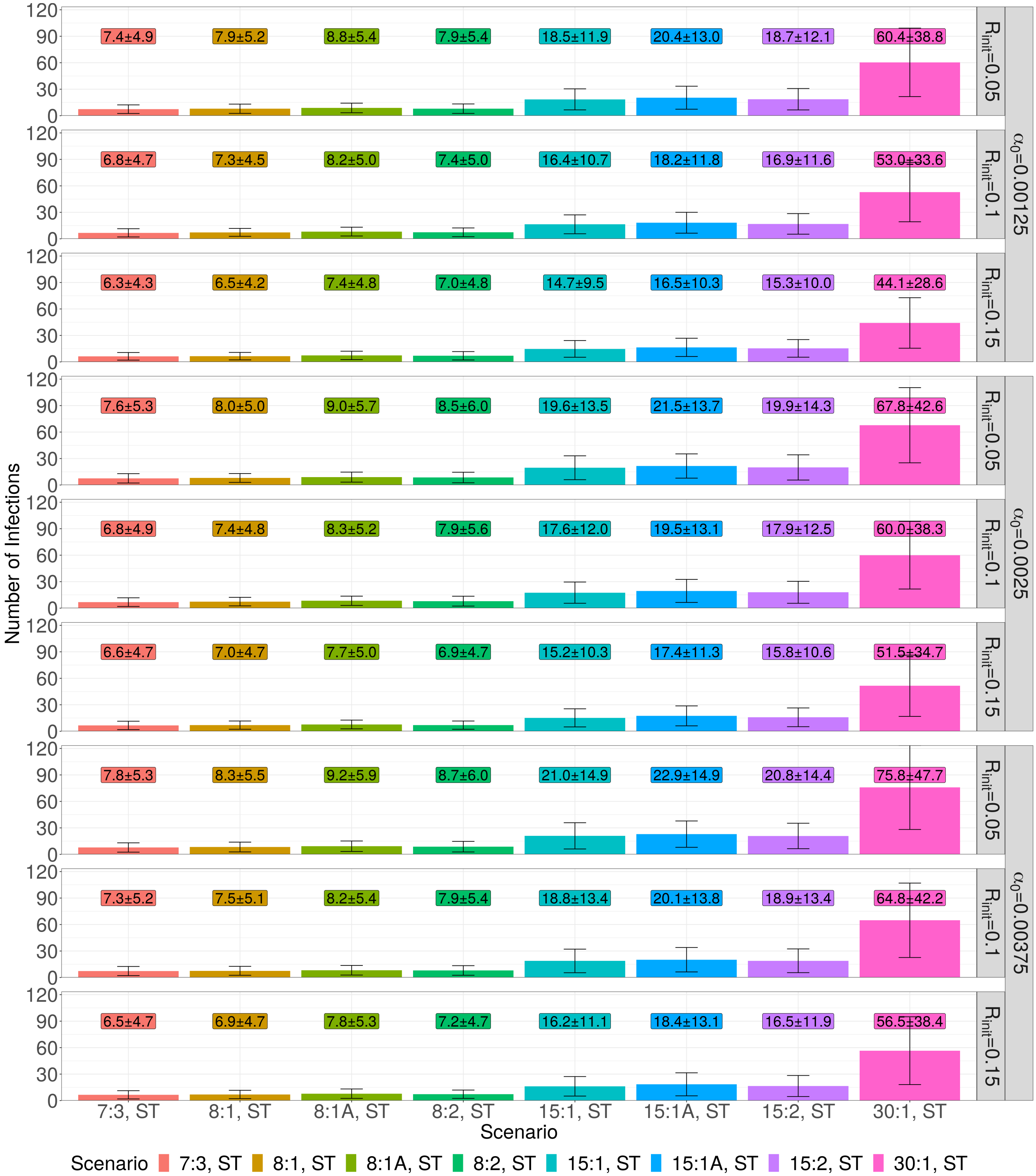

Figure S9. Results of varying the parameters R_{init} and α_0 by (50% each) on the total number of infections for ST assignment. Text in boxes denotes the mean and standard deviation of the data corresponding to the parameters and error bars denote a single standard deviation of the data used.

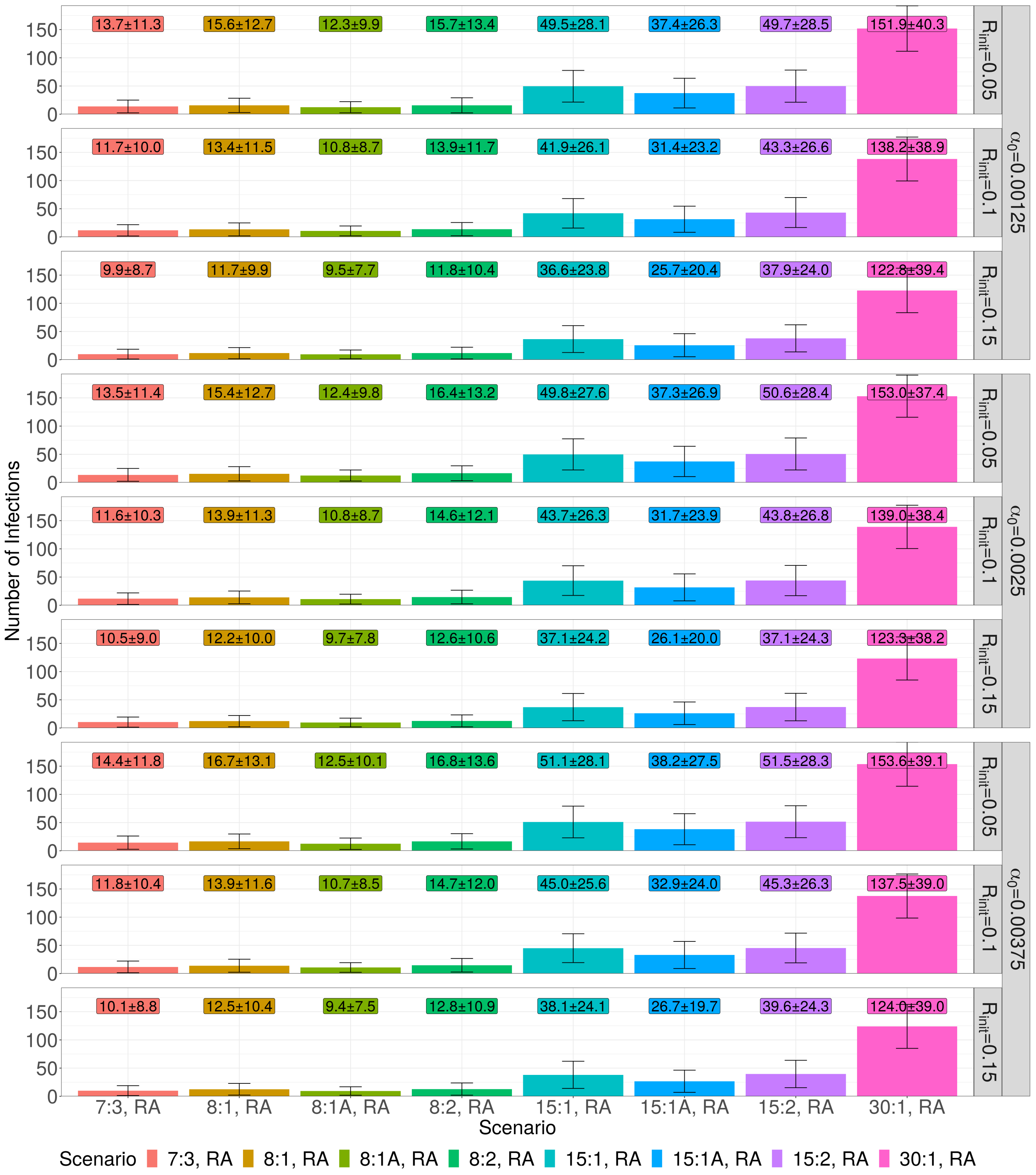

Figure S10. Results of varying the parameters R_{init} and α_0 by (50% each) on the total number of infections for RA assignment. Text in boxes denotes the mean and standard deviation of the data corresponding to the parameters and error bars denote a single standard deviation of the data used.

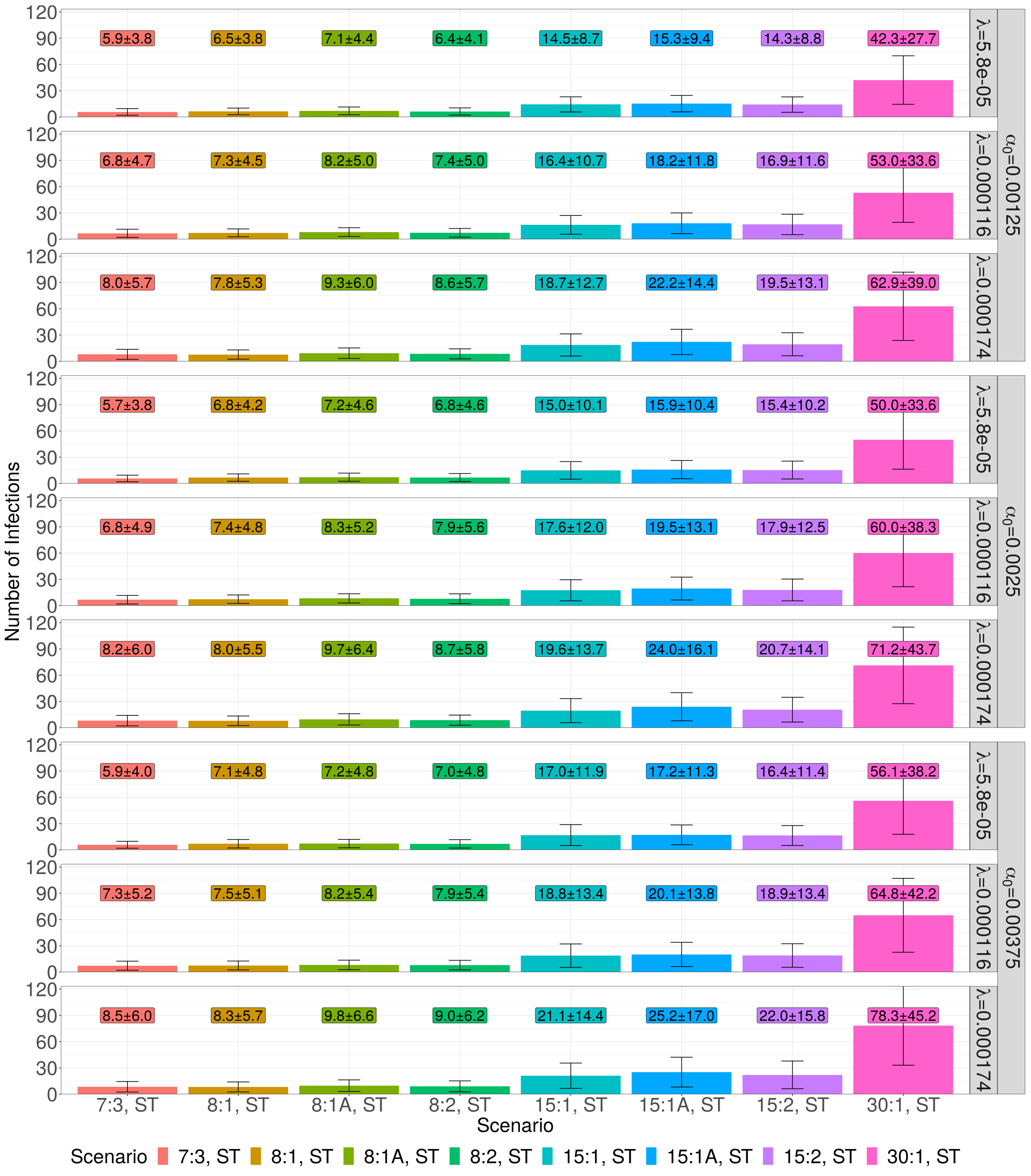

Figure S11. Results of varying the parameters λ_i and α_0 by (50% each) on the total number of infections for ST assignment. Text in boxes denotes the mean and standard deviation of the data corresponding to the parameters and error bars denote a single standard deviation of the data used.

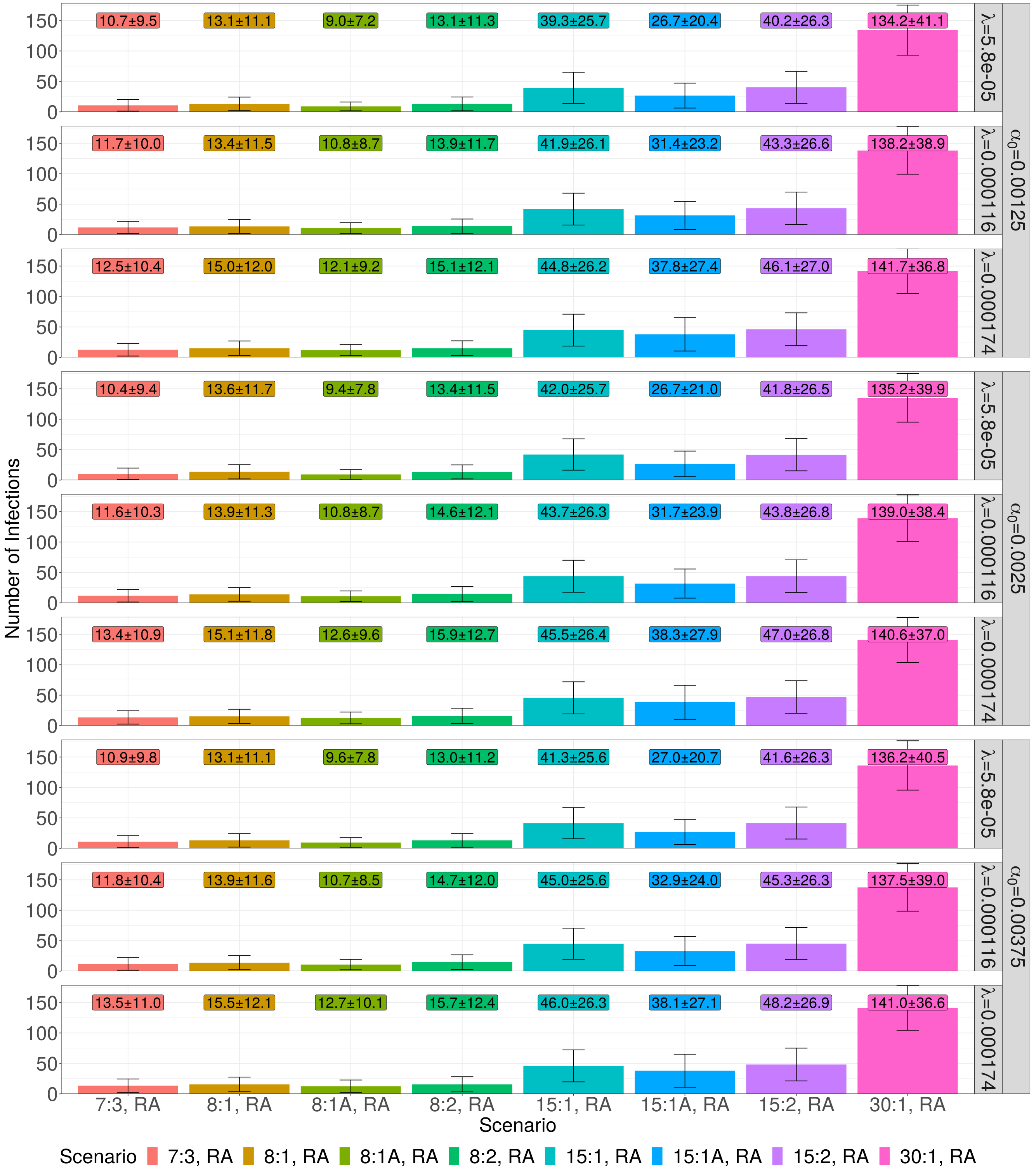

Figure S12. Results of varying the parameters λ_i and α_0 by (50% each) on the total number of infections for ST assignment. Text in boxes denotes the mean and standard deviation of the data corresponding to the parameters and error bars denote a single standard deviation of the data used.
